# Anthrax Edema and Lethal Toxins Differentially Target Human Lung and Blood Phagocytes

**DOI:** 10.3390/toxins12070464

**Published:** 2020-07-20

**Authors:** Vineet I. Patel, J. Leland Booth, Mikhail Dozmorov, Brent R. Brown, Jordan P. Metcalf

**Affiliations:** 1 Department of Medicine, Pulmonary, Critical Care & Sleep Medicine, the University of Oklahoma Health Sciences Center, Oklahoma City, OK 73104, USA; vineet-patel@ouhsc.edu (V.I.P.); john-booth@ouhsc.edu (J.L.B.); Brent-Brown@ouhsc.edu (B.R.B.); 2 Department of Biostatistics, Virginia Commonwealth University, Richmond, VA 23298, USA; mikhail.dozmorov@vcuhealth.org; 3 Department of Microbiology and Immunology, the University of Oklahoma Health Sciences Center, Oklahoma City, OK 73104, USA; 4 Veterans Affairs Medical Center, Oklahoma City, OK 73104, USA

**Keywords:** anthrax, lethal toxin, edema toxin, spores, pathogenesis, human lung, macrophage(s), dendritic cell(s), phagocyte(s), macropinocytosis

## Abstract

*Bacillus anthracis*, the causative agent of inhalation anthrax, is a serious concern as a bioterrorism weapon. The vegetative form produces two exotoxins: Lethal toxin (LT) and edema toxin (ET). We recently characterized and compared six human airway and alveolar-resident phagocyte (AARP) subsets at the transcriptional and functional levels. In this study, we examined the effects of LT and ET on these subsets and human leukocytes. AARPs and leukocytes do not express high levels of the toxin receptors, tumor endothelium marker-8 (TEM8) and capillary morphogenesis protein-2 (CMG2). Less than 20% expressed surface TEM8, while less than 15% expressed CMG2. All cell types bound or internalized protective antigen, the common component of the two toxins, in a dose-dependent manner. Most protective antigen was likely internalized via macropinocytosis. Cells were not sensitive to LT-induced apoptosis or necrosis at concentrations up to 1000 ng/mL. However, toxin exposure inhibited *B. anthracis* spore internalization. This inhibition was driven primarily by ET in AARPs and LT in leukocytes. These results support a model of inhalation anthrax in which spores germinate and produce toxins. ET inhibits pathogen phagocytosis by AARPs, allowing alveolar escape. In late-stage disease, LT inhibits phagocytosis by leukocytes, allowing bacterial replication in the bloodstream.

## 1. Introduction

*Bacillus anthracis* is a Gram positive, spore-forming, rod-shaped, and facultatively anaerobic bacterial species. *B. anthracis* is the causative agent of the disease anthrax, with three forms of the disease occurring depending on the portal of entry of dormant bacterial spores [1]. The majority of anthrax cases occur as cutaneous anthrax, in which spores enter an open wound. A second form, gastrointestinal anthrax, occurs when foods contaminated with spores are ingested and make it to the intestinal tract. The third form of the disease, inhalation anthrax, occurs when dormant spores are inhaled and deposit in the deeper regions of the lung. Inhalation anthrax in humans has a mortality rate greater than 90% if not diagnosed and treated early [1]. This high fatality rate, along with the ease of dissemination of *B. anthracis* spores, has led to this organism being classified as a category A bioterrorism agent (https://www.selectagents.gov/). Events, such as the 2001 letter attacks in the United States [2] and the 1979 Sverdlovsk accident in the former Soviet Union [3], highlight the real-world dangers of its use as a bioterrorism weapon. 

*B. anthracis* spores are a dormant form of the bacterium with an average diameter of 1–1.5 μm [4]. The diameter of the opening to the alveoli of the human lung is about 5 μm [5], which allows the deposition of spores within these gas exchange structures. It is well-established that bacterial dissemination occurs unidirectionally from the alveoli to the mediastinal lymph nodes (mLNs), and then to the bloodstream, resulting in septicemia [6,7]. However, the means of alveolar escape during the early stages of human infection remain a mystery. Four mechanisms of alveolar escape have been proposed. The first involves macrophages (MΦ) serving as a carrier cell or a “Trojan horse” that migrates to the mLN with internalized dormant and/or germinating spores [8]. Various studies, including our work, have shown that MΦ rapidly internalize *B. anthracis* spores [8,9,10]. Against this possibility is the fact that MΦ do not express C-C chemokine receptor 7 (CCR7), which is implicated in migration toward lymph nodes from the periphery [11,12]. The second hypothesis suggests that dendritic cells (DCs) are the Trojan horse for *B. anthracis* spores [13]. Although these cells are of low frequency in the alveoli, they are known to internalize spores, express CCR7, and migrate to the mLN [13,14,15]. A third mechanism posits that spores do not need a carrier cell at all but rather that they are transported transcellularly from the apical to the basolateral side of the polarized alveolar epithelium [16,17,18]. Once across the epithelium, the spores can enter lymphatic vessels and reach the lymph nodes, where they then germinate. The fourth mechanism suggests that some spores germinate locally within the alveoli and begin producing virulence factors [19]. These virulence factors help to subdue innate immune cells within and along the alveoli, and also break down the epithelial barrier so that spores and vegetative bacteria can access the lymphatics. This means of escape has been termed the “jailbreak” model, based on the proposed mass escape of the pathogen once the alveolar epithelium is compromised [19]. Current evidence has not eliminated any of these potential mechanisms during early stages of inhalation anthrax in humans, and it is also possible that multiple mechanisms of alveolar escape occur simultaneously.

Vegetative *B. anthracis* produces three main virulence factors: (1) An antiphagocytic poly-D-γ-glutamic acid capsule; (2) lethal toxin (LT), which is a zinc-dependent metalloproteinase that cleaves cellular mitogen-activated protein kinases (MEKs); and (3) edema toxin (ET), which is a calmodulin-dependent adenylyl cyclase that dramatically increases intracellular cyclic adenosine monophosphate (cAMP) levels [20,21,22,23]. The two exotoxins, LT and ET, are classic A-B bacterial toxins [1]. LT is a combination of lethal factor (LF) and protective antigen (PA), with LF containing metalloproteinase activity and PA serving as the cellular binding component. ET is a combination of edema factor (EF) and PA, with EF being an adenylyl cyclase and PA again serving as the binding component. Vegetative bacilli produce and secrete PA, LF, and EF soon after spore germination [24]. Secreted PA is an 83 kDa protein, which is cleaved by cell surface or circulating proteases into PA_20_ and PA_63_ components [25,26]. PA_63_ oligomerizes into heptamers or octamers, with each oligomer capable of binding three or four LF and EF molecules [27,28]. PA has two known cellular receptors: tumor endothelium marker-8/anthrax toxin receptor 1 (TEM8/ANTXR1) and capillary morphogenesis protein-2/anthrax toxin receptor 2 (CMG2/ANTXR2) [29,30]. The physiological function(s) of these receptors remain unclear. With regards to PA, CMG2 has an 11-fold higher affinity for PA than TEM8 [31]. This is reflected in vivo, where mice lacking CMG2 are resistant to LT-induced lethality and cutaneous spore challenge, while mice lacking TEM8 are susceptible to both types of challenge [31]. Additionally, data from a large human cohort suggests that, at least with B cells, 11–24% of the variation in anthrax toxin sensitivity is accounted for by the level of CMG2 mRNA [32]. Whether this correlation is maintained at the protein level or whether it appears in other human cell types remains unknown. PA oligomerizes after binding to TEM8/CMG2, or the entire oligomer binds at one time [27,28]. In either case, the oligomer with bound LF and/or EF is then internalized via receptor-mediated endocytosis [27,33,34]. As the endosome acidifies, LF and EF detach from PA, and the oligomer itself becomes a pore through which the two active components enter the cytoplasm to exert their intracellular toxic effects [35,36]. 

LT causes rapid cell death in the mouse macrophage cell lines RAW 264.7 [37] and J774 [38]. Primary MΦ isolated from BALBc mouse [39] and Fischer rat strains [40] show a similar sensitivity to LT. This observed lethality originally made alveolar macrophages (AMs) a likely target of LT-induced death during the early stages of inhalation anthrax, perhaps inhibiting the ability of AM to destroy the pathogen before transit to mLN. However, our group determined that, unlike these rodent cell lines and in vivo models, primary human AM are resistant to the effects of LT at levels as high as 1000 ng/mL [41]. They express minimal amounts of TEM8 and no detectable protein level of CMG2. Furthermore, human AMs do not undergo MEK cleavage or induction of apoptosis in response to LT exposure. These results suggested that AMs are actually a major sentinel cell against early *B. anthracis* exposure in the human lung due to their resistance to LT [41] and their ability to internalize *B. anthracis* spores [9,10].

While AMs are the highest frequency phagocytic cell type in the human alveoli, they are not the only type of phagocyte found there under resting conditions [10,15]. Aside from AMs, we recently identified and classified five other subsets of airway and alveolar resident phagocytes (AARPs) in the human lung based on surface marker expression and whole-genome transcriptional profiling [10]. These six phagocyte subsets can be grouped into MΦ/monocyte and DC clades based on these profiles. AM, BDCA1− CD14+ cells, and BDCA1− CD14− cells fall into the MΦ/monocyte clade, while Langerin+, BDCA1+ CD14+, and BDCA1+ CD14− cells are part of the DC clade. Other groups have also identified similar phagocyte subsets in the human lung, though with slightly different marker schema [15]. We also determined that all six AARP subsets internalize bacterial particles, including *Staphylococcus aureus*, *Escherichia coli*, and *B. anthracis* spores, within 30 min of exposure [10]. In relation to *B. anthracis* spores, AM and BDCA1− CD14+ cells are especially efficient at spore uptake [10]. 

Under the “jailbreak” model of anthrax pathogenesis, some *B. anthracis* spores germinate locally in the alveoli and begin secreting PA, LF, and EF [19]. The six AARP subsets we previously characterized within and along the alveoli would be exposed to LT and ET under this model. We already showed that AMs are resistant to LT-induced cytotoxicity [41], but the effects of LT on the other five subsets remain untested. In the current study, we examined RAW 264.7 cells (as a cellular control known to be susceptible to LT), primary human AARP subsets, and primary human leukocytes, for their responses to LT and ET. Specifically, we measured TEM8/CMG2 expression on and PA binding by these cells, along with induction of apoptosis/necrosis and *B. anthracis spore* internalization after toxin exposures at varying concentrations. We hypothesized that at least one of the AARP subsets would demonstrate susceptibility to anthrax toxins, and thus would provide insight into the likely mechanism(s) of alveolar escape during the early stages of inhalation anthrax. 

We measured low surface expression of TEM8 and CMG2 by human AARPs and leukocytes by flow cytometry. All tested cell types bound PA in a dose-dependent manner. LT did not induce apoptosis or necrosis in any of the AARP subsets or leukocyte populations within 3 h of toxin exposure. However, toxin preincubation significantly decreased spore uptake. In AARPs, this effect was caused predominantly by ET. In both phagocytic and non-phagocytic leukocytes, LT was the major effector responsible for decreased spore internalization. Collectively, these results could implicate ET in helping *B. anthracis* escape from the alveoli early in infection by preventing spore phagocytosis and subsequent destruction by AARPs. In later stages of disease, LT at high levels in the bloodstream may induce global inhibition of particle internalization by leukocytes. This effect may be the principal mechanism of LT virulence, allowing *B. anthracis* to replicate to high numbers in the blood. 

## 2. Results

We previously showed that human AMs are resistant to the effects of *B. anthracis* LT [41]. Human AM express minimal levels of the anthrax toxin receptors ANTXR1/TEM8 and no measurable levels of ANTXR2/CMG2 protein, and are not susceptible to LT-induced apoptosis or LT-mediated MEK cleavage. Our group recently classified and characterized five additional AARP subsets from the resting human lung [10]. These rarer cell subsets can be grouped into MΦ and DC clades based on their transcriptional profiles and functional characteristics. As a logical next step, we sought to determine if these rarer AARP subsets were sensitive to anthrax LT, as well as ET. 

### 2.1. Small Percentages of Human AARP Subsets Express Surface TEM8 and CMG2

**Figure 1:**
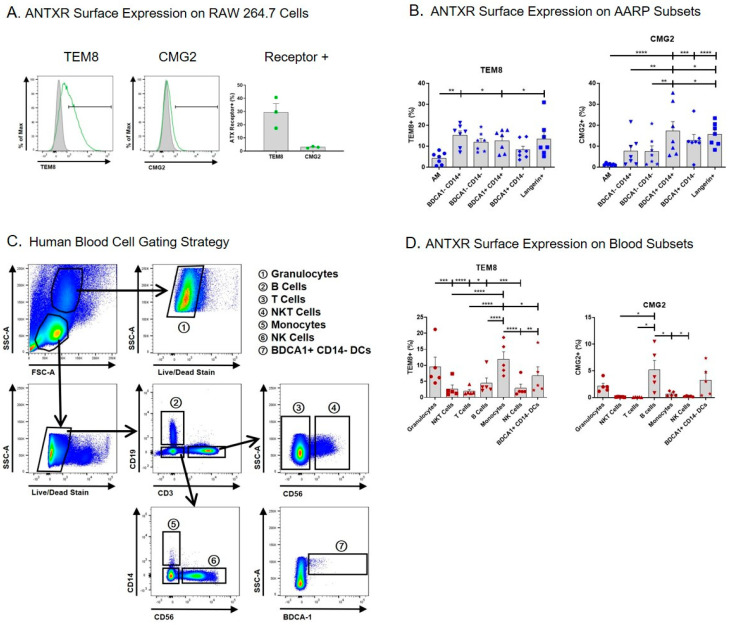
Less than 20% of human AARP subsets express surface TEM8 and CMG2. (**A**) RAW 264.7 cells analyzed by flow cytometry for TEM8 and CMG2 surface expression. Histograms depict TEM8 (left) and CMG2 (middle) positivity (gates shown) measured by flow cytometry of cultured RAW 264.7 cells. Representative plots from 3 independent experiments are shown. Gray, isotype control. Right, percentages of RAW 264.7 cells positive for surface TEM8 and CMG2 expression. Composite data are expressed as mean + SEM with individual results from 3 independent experiments overlaid (green). (**B**) Percentages of each of the 6 AARP subsets positive for surface TEM8 (left) and CMG2 (right) expression. Composite data as mean + SEM, with individual results from 7 lung donor overlaid (blue) shown. (**C**) Flow cytometry of human leukocytes from buffy coats. Gating strategy used to identify 7 subsets (labeled 1-7 above). After exclusion of dead cells, populations of (1) granulocytes, (2) B cells, (3) T cells, (4) NKT cells, (5) monocytes, (6) NK cells, and (7) BDCA1+ CD14- DCs were identified. Representative plots from 5 blood donors are shown. (**D**) Percentages of each of the 7 blood subsets positive for surface TEM8 (left) and CMG2 (right) expression. Composite data are mean + SEM, with individual results from 5 blood donors overlaid (red) shown. * *p* ≤ 0.05, ** *p* ≤ 0.01, *** *p* ≤ 0.001, **** *p* ≤ 0.0001, One-way ANOVA with Tukey’s multiple comparisons test.

We began by measuring the surface expression of TEM8 and CMG2 on the AARP subsets, and also human leukocytes, by flow cytometry (Figure 1). We used the mouse MΦ cell line RAW 264.7 as a positive control, as this line is known to express TEM8 and CMG2 mRNA and protein [41,42,43,44]. An average of 30% of RAW 264.7 cells expressed surface TEM8, while only ~3% of these cells expressed surface CMG2 compared to isotype controls (Figure 1A). Using cells from seven lung donors, we measured the expression of TEM8 and CMG2 on the surface of the six AARP subsets. Consistent with our previous work [41], AM displayed the lowest percentage of TEM8 positivity compared to the other five subsets (Figure 1B left). There were no other statistical differences or trends between the other subsets. In all cases, less than 20% of the subsets were positive for TEM8. CMG2 positivity was similarly below 20% across all six AARP subsets, although AM positivity was statistically the lowest (less than 2%) (Figure 1B right). The AM results are consistent with our previous work suggesting CMG2 protein levels in AM were negligible [41]. Langerin+ and BDCA1+ CD14+ cells did have statistically higher CMG2 positivity compared to the other four AARP subsets. Aside from AM, CMG2 positivity was greater in AARP subsets compared to RAW 264.7 cells. Overall, surface expression of both toxin receptors was low on all AARP subsets.

As blood cells are also exposed to anthrax toxins during infection [45], we also analyzed the cell surface positivity of TEM8 and CMG2 on human leukocytes (Figure 1C,D). Seven leukocyte populations were consistently identified based on a modified gating strategy [46]: Granulocytes (mostly neutrophils), B cells, T cells, NKT cells, monocytes, NK cells, and BDCA1+ CD14− DCs (Figure 1C). These cells were not further split into subsets, such as T helper cells and cytotoxic T cells, as this was beyond the scope of this study. Analyses of blood BDCA1+ CD14− DCs were included for comparison to the effects measured on BDCA1+ CD14− cells from the lung. The percent positivity of TEM8 was less than 15% for all leukocytes, with phagocytic cells (granulocytes, monocytes, and BDCA1+ CD14− DCs) showing significantly more positivity than the non-phagocytic cell types (NKT cells, T cells, B cells, and NK cells) (Figure 1D left). CMG2 positivity was 5% or less for all leukocytes, with only granulocytes, B cells, and BDCA1+ CD14− DCs displaying more than 1% mean positivity (Figure 1D right). These results collectively suggest that, at least using flow cytometry, the majority of each of the six human AARP subsets and seven leukocyte populations we examined do not express surface TEM8 or CMG2.

### 2.2. B. anthracis PA Binds to Human AARP Subsets in a Dose-dependent but ANTXR-independent Manner

**Figure 2:**
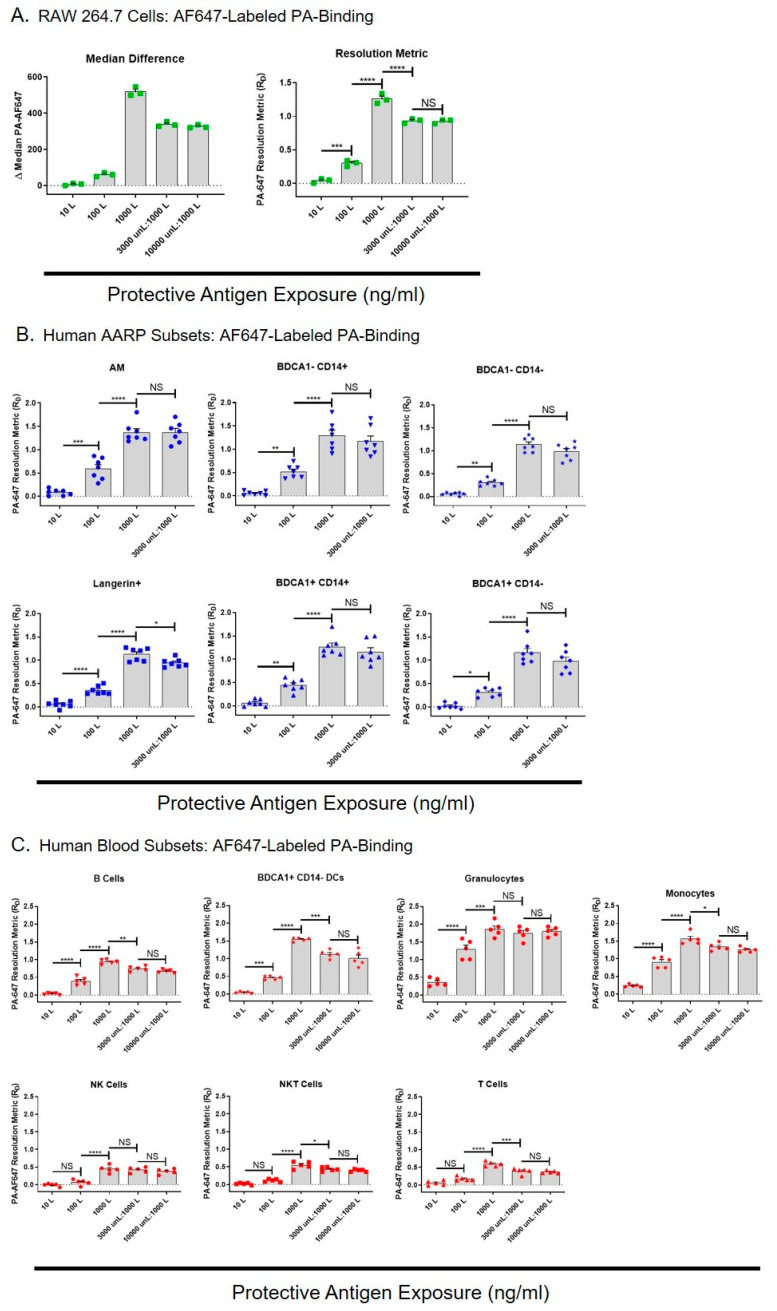
PA binds to human AARP subsets in a dose-dependent but ANTXR-independent manner. (**A**) AlexaFluor647-labeled PA binding to RAW 264.7 cells as depicted by the median difference compared to the untreated (left) and resolution metric (right). Cells were exposed to 10, 100, or 1000 ng/mL AF647-PA for 30 min, or pre-incubated with unlabeled PA at 3000 and 10,000 ng/mL for 30 min prior to AF647-PA exposure at 1000 ng/mL. Graphs represent mean + SEM with results from 3 independent experiments overlaid (green). (**B**) AF647-PA binding to AARP subsets as measured by resolution metrics. BAL cells were exposed to 10, 100, or 1000 ng/mL AF647-PA for 30 min, or preincubated with unlabeled PA at 3000 ng/mL for 30 min prior to AF647-PA exposure at 1000 ng/mL. Graphs represent mean + SEM with individual results from 7 lung donors overlaid (blue). (**C**) AF647-PA binding to blood subsets as measured by resolution metrics. Blood cells were exposed to 10, 100, or 1000 ng/mL AF647-PA for 30 min, or pre-incubated with unlabeled PA at 3000 or 10,000 ng/mL for 30 min prior to AF647-PA exposure at 1000 ng/mL. Graphs represent mean + SEM with results from 5 blood donors overlaid (red). L=Labeled PA, unL=unlabeled PA. NS = not significant, * *p* ≤ 0.05, ** *p* ≤ 0.01, *** *p* ≤ 0.001, **** *p* ≤ 0.0001, One-way ANOVA with Tukey’s multiple comparisons test.

We next wanted to determine if the TEM8 and CMG2 on the surface of the AARP subsets was biologically active, and also whether anthrax toxins could enter these cells independently of toxin receptors. To measure this, we examined the binding of dye-labeled PA to RAW 264.7 cells, human AARPs, and human leukocytes (Figure 2). To compare PA binding between these different cell types, each having different frequencies, population spreads, and autofluorescence levels, we converted fluorescence differences measured by flow cytometry into resolution metrics (R_D_). The resolution metric normalizes highly variable flow cytometry results by correcting for both the distance and the spread of the data [47,48,49]. R_D_ is defined as: (median_test_ − median_control_)/(rSD_test_ + rSD_control_), where median is the median fluorescence intensity and rSD is the robust standard deviation of a cell population under test or control conditions. It is used to measure marker expression-level data (rather than just positive or negative), and since PA binding is based on receptor expression, R_D_ was a useful way of allowing comparison between the cell types. RAW 264.7 cells are sensitive to LT-induced lethality [37,44], so we expected that they would bind PA. RAW 264.7 cells were exposed to 10,100, or 1000 ng/mL AF647-labeled PA for 30 min, or preincubated with 3000 or 10,000 ng/mL unlabeled PA for 30 min prior to exposure to labeled PA at 1000 ng/mL. We compared the median difference values (difference in median values compared to untreated cells) to resolution metrics, and saw the same patterns of results with increasing PA exposure concentrations (Figure 2A left and right). This similarity between the two means of data representation verified the use of R_D_ for further comparisons between cell types. As expected, PA binding statistically increased with increasing AF647-PA exposure. When we preincubated with unlabeled PA at 3X the amount of labeled PA, it statistically decreased the AF647-PA bound (Figure 2A right). However, labeled PA binding did not further decrease it if we preincubated RAW 264.7 cells with unlabeled PA at 10X the amount of labeled PA. This suggested that PA was binding or being internalized by RAW 264.7 cell via an additional mechanism that was not saturable.

We then performed the same AF647-PA exposures with the six human AARP subsets (Figure 2B). Labeled PA binding significantly increased in all AARPs with increasing doses of 10, 100, and 1000 ng/mL. PA binding was similar across AARP subsets at all concentrations, although it appeared slightly higher in AM and BDCA1− CD14+ cells at the 100 ng/mL exposure. Preincubating these cells with 3000 ng/mL unlabeled PA did not significantly decrease AF647-PA binding except in Langerin+ cells. This suggested that in AARPs, labeled PA was mostly binding to the cell surface via alternate receptors that were not saturated by the unlabeled PA, and/or was being internalized by non-specific mechanisms like macropinocytosis. As we saw that 10,000 ng/mL unlabeled PA did not further decrease labeled PA binding of RAW 264.7 cells (Figure 2A), we did not use this higher concentration pretreatment on AARPs. We also wanted to measure PA binding on human leukocytes to determine how their toxin interactions compared to those of AARP subsets (Figure 2C). Again, increasing AF647-PA exposure levels increased PA binding from 10 ng/mL to 1000 ng/mL. Phagocytic leukocytes, specifically granulocytes, monocytes, and BDCA1+ CD14− DCs, bound/internalized more labeled PA compared to the non-phagocytic B cells, NK cells, NKT cells, and T cells. These observations were particularly dramatic at the 100 and 1000 ng/mL PA exposures. Unlike the AARP subsets, but similar to RAW 264.7 cells, most of the leukocytes showed significantly decreased AF647-PA binding when preincubated with 3000 ng/mL unlabeled PA. However, this interaction did not further decrease after 10,000 ng/mL unlabeled PA exposure. Exceptions were granulocytes and NK cells, which did not display decreased PA binding even with the 3000 ng/mL unlabeled PA preincubation. 

In order to compare the PA binding potential of RAW 264.7 cells, the AARP subsets, and the leukocytes, we compared their R_D_ values at the different AF647-PA exposure levels (Figure S1). Even at the low-dose PA exposure of 10 ng/mL, granulocytes and monocytes demonstrated significantly greater binding than all the other cell types (Figure S1 Top). For granulocytes, this remained the case at 100 and 1000 ng/mL exposures (Figure S1 middle and bottom). Conversely, at 1000 ng/mL, T cells, NKT cells, and NK cells showed significantly lower PA binding (Figure S1 bottom). Interestingly, BDCA1+ CD14− DCs from the blood bound/internalized significantly more PA that their lung counterparts at 1000 ng/mL exposure. RAW 264.7 cells and the AARP subsets showed similar PA binding across all exposure levels. Together, these results suggest that PA can bind and/or be internalized by human AARPs and leukocytes via one or more mechanisms that are independent of TEM8 and CMG2 surface expression. Additionally, phagocytic cells of the lung and blood bind/internalize more PA than non-phagocytic leukocytes.

### 2.3. Human AARP Subsets are Resistant to LT-induced Apoptosis and Necrosis

**Figure 3:**
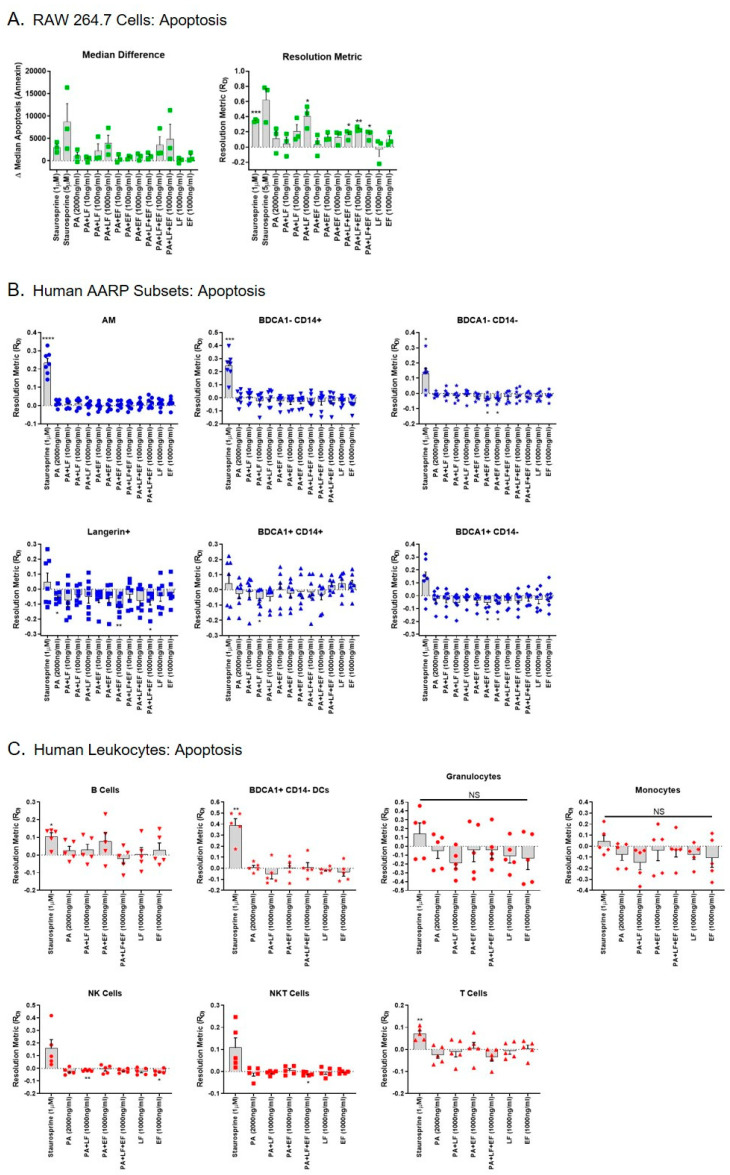
Anthrax lethal toxin does not induce apoptosis in human AARP subsets or leukocytes. (**A**) Apoptosis of RAW 264.7 cells as median differences (left) and resolution metrics (right) compared to untreated cells. Cells were exposed to 10, 100, or 1000 ng/mL LT, ET, or LT+ET for 3 h, followed by staining with PE-Annexin V. Staurosporine at 1 and 5 μM served as positive controls. Graphs represent mean + SEM with results from 3 independent experiments overlaid (green). (**B**) Apoptosis of AARP subsets as measured by resolution metrics. BAL cells were exposed to Staurosporine (1 μM), or 10, 100, or 1000 ng/mL, LT, ET, or LT+ET for 3 h, followed by Annexin V staining. Graphs represent mean + SEM with individual results from 7 lung donors overlaid (blue). (**C**) Apoptosis of blood subsets as measured by resolution metrics. Blood cells were exposed to Staurosporine (1 μM) or 1000 ng/mL, LT, ET, or LT+ET for 3 h, followed by Annexin V staining. Graphs represent mean + SEM with individual results from 5 blood donors overlaid (red). NS = not significant, * *p* ≤ 0.05, ** *p* ≤ 0.01, *** *p* ≤ 0.001, **** *p* ≤ 0.0001, One-sample t test of each treatment to untreated control.

Previous work has shown that LT induces apoptosis in the mouse MΦ RAW 264.7 cell line [37]. However, we previously demonstrated that human AMs are resistant to LT-induced apoptosis after 3 h of toxin exposure [41]. We next determined if any of the rarer AARP subsets were susceptible to LT-induced apoptosis in this same time frame. If so, this could suggest a mechanism by which *B. anthracis* impairs the innate immune response during early stages of inhalation anthrax in the lung. We first exposed RAW 264.7 cells to increasing concentrations (10, 100, or 1000 ng/mL) of LT, ET, or LT and ET (Figure 3A). Staurosporine at 1 and 5 μM was used as a positive control for induction of apoptosis. We then measured apoptosis based on the cell binding of dye-labeled Annexin V. Again, we plotted the results as median differences and resolution metrices, with similar patterns being observed using the two methods (Figure 3A). As expected, 1 μM staurosporine significantly induced apoptosis in RAW 264.7 cells compared to no treatment (zero value). LT also significantly induced apoptosis at the 1000 ng/mL exposure compared to no treatment, as did LT plus ET at 10, 100, and 1000 ng/mL (Figure 3A right). None of the treatments were significantly different from each other in RAW 264.7 cells when compared by repeated measures one-way ANOVA. As expected, ET alone did not induce apoptosis in these cells. However, the combination of LT and ET induced apoptosis at concentrations of 10 and 100 ng/mL. This occurred even though PA was included at 2x the amount of LF or EF alone (i.e., PA was included at 2000 ng/mL when the combined treatment with LF and EF at 1000 ng/mL occurred). Collectively, these results show that LT induces apoptosis in RAW 264.7 cells within 3 h, but ET may exert toxic effects on RAW 264.7 cells in the presence of LT. This effect is not obvious in the magnitude of apoptosis, as LT alone at 1000 ng/mL induces more apoptosis than LT/ET at the same concentration. Rather, it is in the fact that LT alone at 10 or 100 ng/mL does not significantly increase apoptosis, while LT with ET at these concentrations does. 

We next exposed the human AARP subsets to the same treatments, except 5 μM staurosporine was not used to induce apoptosis in AARPs (Figure 3B). Exposure to 1 μM staurosporine was enough to significantly induce apoptosis in the three macrophage/monocyte clade subsets (AM, BDCA1− CD14+ cells, and BDCA1− CD14− cells) (Figure 3B top). The DC clade members displayed a more biphasic response to staurosporine, with some lung donors’ cells displaying resistance to apoptosis after 3 h exposure (Figure 3B bottom). Interestingly, LT at concentrations as high as 1000 ng/mL did not induce apoptosis in any of the AARP subsets after 3 h of toxin exposure. This absence of apoptosis induction also occurred with coexposure to equal concentrations of ET. ET exposure alone actually significantly decreased apoptosis in BDCA1− CD14−, Langerin+, and BDCA1+ CD14− AARP subsets compared to untreated cells. We also wanted to compare our AARP results to those in human leukocytes to determine if any of these seven cell types were sensitive to LT-induced apoptosis (Figure 3C). As expected, 1 μM staurosporine induced apoptosis in a majority of leukocytes. However, LT treatment did not cause apoptosis in any of the leukocytes at a concentration of 1000 ng/mL after 3 h of exposure. We excluded the lower concentration toxin exposures after measuring no significant effect of these levels of LT and ET in human AARPs. ET alone or in combination with LT at 1000 ng/mL did not cause or enhance apoptosis in the leukocytes. Finally, we compared the resolution metrics of apoptosis for LT, ET, and LT plus ET at 1000 ng/mL for all cells (Figure S2). Only RAW 264.7 cells exhibited LT-induced apoptosis. These cells were sensitive to LT alone or to LT and ET together, while the AARP subset and leukocyte cell types were not (Figure S2 top and bottom). Together, these results show that, unlike the model RAW 264.7 cell line, human AARP subsets and leukocytes are resistant to LT-induced apoptosis at concentrations up to 1000 ng/mL and exposure times up to 3 h.

**Figure 4:**
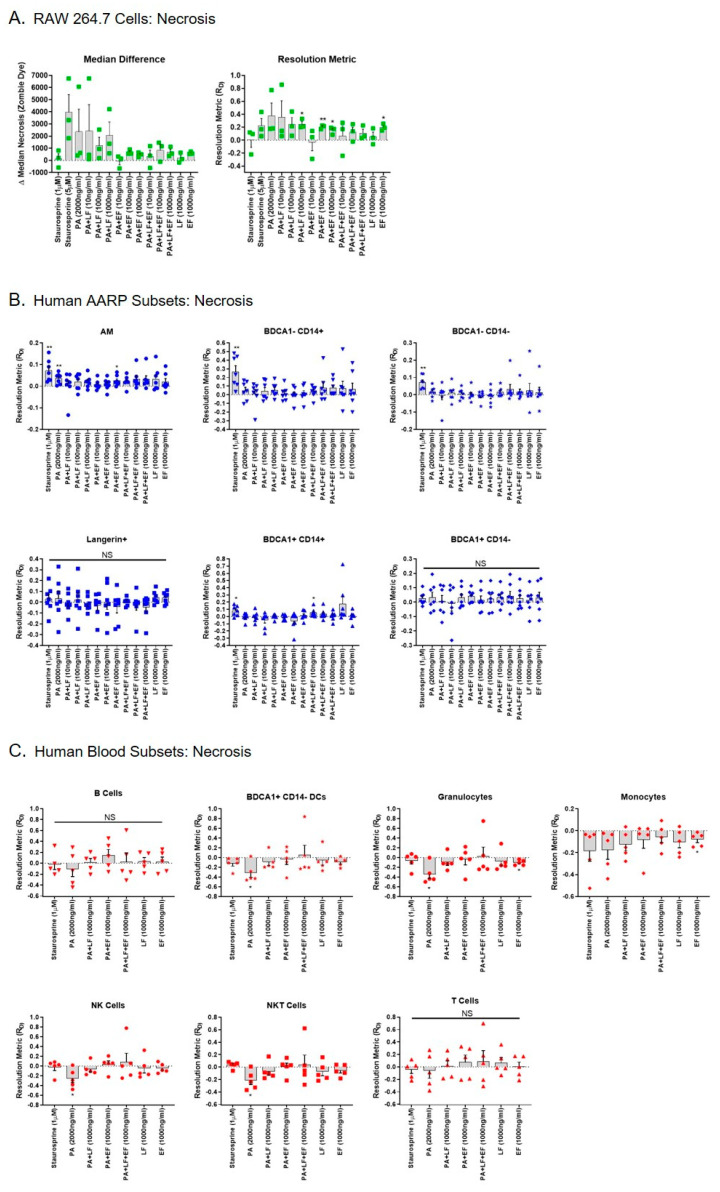
Anthrax LT and ET induce necrosis in RAW 264.7 cells but not in human AARP subsets. (**A**) Necrosis of RAW 264.7 cells as the median differences (left) and resolution metric (right) compared to untreated cells. Cells were exposed to 10, 100, or 1000 ng/mL LT, ET, or LT+ET for 3 h, followed by staining with Zombie Aqua viability dye (necrosis). Graphs represent mean + SEM with results from 3 independent experiments overlaid (green). (**B**) Necrosis of AARP subsets as measured by resolution metrics. BAL cells were exposed to Staurosporine (1 μM), or 10, 100, or 1000 ng/mL, LT, ET, or LT+ET for 3 h, followed by Zombie Aqua staining. Graphs represent mean + SEM with individual results from 7 lung donors overlaid (blue). (**C**) Necrosis of blood subsets as measured by resolution metrics. Blood cells were exposed to Staurosporine (1 μM) or 1000 ng/mL, LT, ET, or LT+ET for 3 h, followed by Zombie Aqua staining. Graphs represent mean + SEM with individual results from 5 blood donors overlaid (red). NS = not significant, * *p* ≤ 0.05, ** *p* ≤ 0.01, One-sample *t* test of each treatment to untreated control.

As LT has been shown to also induce necrosis in certain cell models [38,50,51], we also tested whether LT or ET induced necrosis in RAW 264.7 cells, human AARPs, or human leukocytes (Figure 4). Staurosporine at 1 and 5 μM concentrations did not increase necrosis in RAW 264.7 cells (Figure 4A). LT at 1000 ng/mL significantly induced necrosis in these cells within 3 h of exposure (Figure 4A). Surprisingly, ET at 100 and 1000 ng/mL also induced necrosis in these cells, as did EF alone at 1000 ng/mL. Unlike the RAW 264.7 cells, four of the six AARP subsets were sensitive to staurosporine-induced necrosis: AM, BDCA1− CD14+ cells, BDCA1− CD14− cells, and BDCA1+ CD14+ cells (Figure 4B). LT did not cause necrosis in any of the human AARP subsets (Figure 4B) or human leukocytes (Figure 4C) at concentrations as high as 1000 ng/mL after 3 h of toxin exposure. ET also did not induce necrosis in a majority of AARPs and leukocytes, although AM did display a significant induction of necrosis after 3 h of ET exposure at 1000 ng/mL and BDCA1+ CD14+ cells showed increased necrosis after LT/ET exposure at 10 ng/mL. We also compared necrosis in RAW 264.7 cells, human AARP subsets, and human leukocytes at the highest exposure levels of LT, ET, or LT plus ET (Figure S3). Compared to human lung and blood cells, LT alone caused greater necrosis of RAW 264.7 cells (Figure 3 top). ET alone appeared to also cause greater necrosis, although not as dramatically (Figure 3 middle). The combination of LT with ET at 1000 ng/mL did not discernably cause necrosis in any of the cell types (Figure 3 bottom). Overall, these results reveal that human AARPs and leukocytes are resistant to LT-induced necrosis at concentrations as high as 1000 ng/mL and exposure times as long as 3 h. As with RAW 264.7 cells, AM and BDCA1+ CD14+ cells may be sensitive to ET-induced necrosis.

### 2.4. Anthrax ET Decreases B. anthracis Spore Internalization by Human AARP Subsets, While LT Decreases Spore Internalization by Human Leukocytes

**Figure 5:**
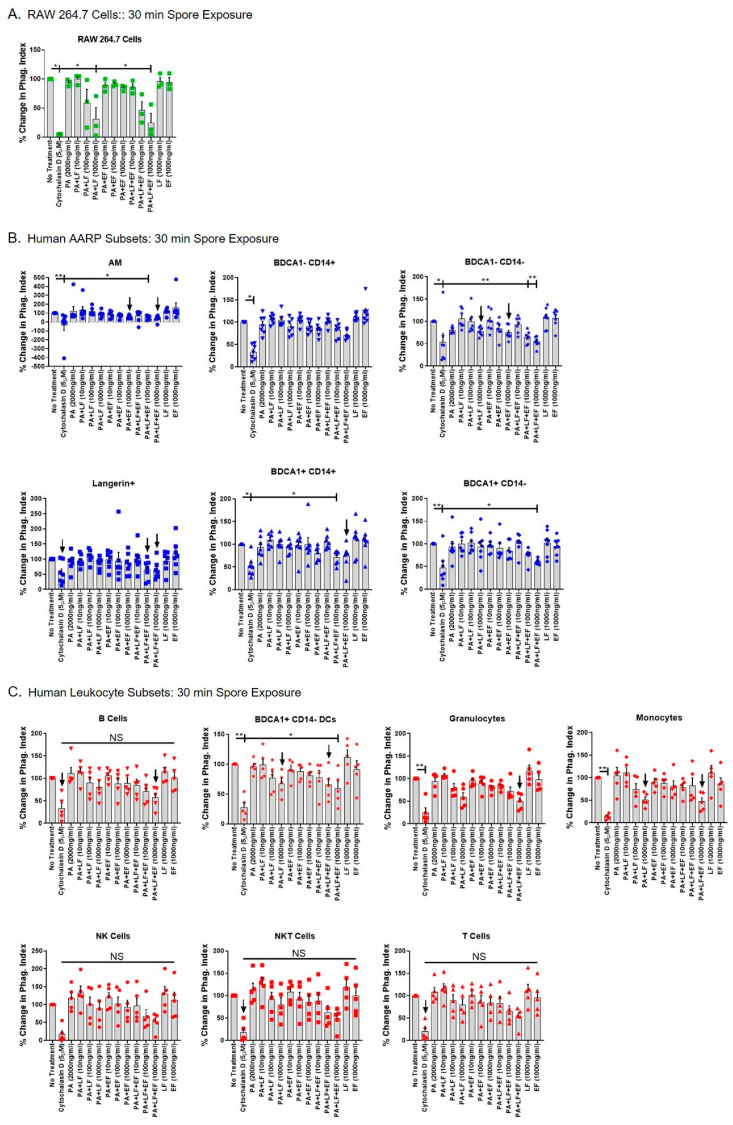
Anthrax toxins decrease early *B. anthracis* spore internalization by human AARP subsets and phagocytic leukocytes. Internalization of pHrodo-labeled *Ba* spores by (**A**) RAW 264.7 cells, (**B**) human AARPs, and (**C**) human leukocyte subsets, after toxin pretreatments. Cells were preincubated with 10, 100, or 1000 ng/mL LT, ET, or LT+ET for 3 h, followed by 30 min of spore exposure at 10 spores/cell. Cytochalasin D (5 μM) pretreatment for 1 h prior to spore addition served as a control for internalization inhibition. Graphs show the percent change in phagocytic indexes (PIs) compared to no treatment. PIs were calculated as the percent of a given cell subset positive for pHrodo signal multiplied by the median fluorescence intensity of the pHrodo-positive cells of that subset. (**A**) % change in PIs of RAW 264.7 cells. Graphs represent mean + SEM with results from 3 independent experiments overlaid (green). (**B**) % change in PIs of human AARP subsets. Graphs represent mean + SEM with individual results from 7 lung donors overlaid (blue). (**C**) % change in PIs of human leukocyte subsets. Graphs represent mean + SEM with individual results from 5 blood donors overlaid (red). NS = not significant, * *p* ≤ 0.05, ** *p* ≤ 0.01, Friedman test with Dunn’s multiple comparisons test to no treatment. Black arrows indicate trends based on *p* values less than 0.20.

**Figure 6:**
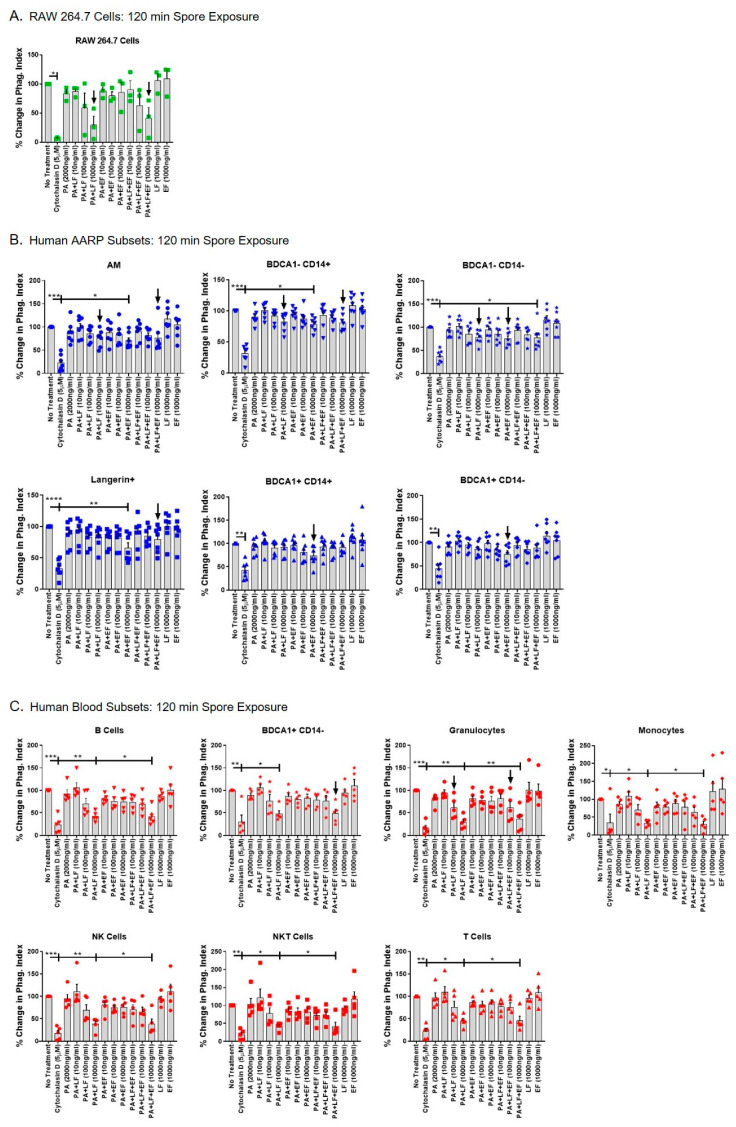
Anthrax ET decreases late *B. anthracis* spore internalization by human AARPs, while LT decreases internalization by leukocytes. Internalization of pHrodo-labeled *Ba* spores by (**A**) RAW 264.7 cells, (**B**) human AARPs, and (**C**) human leukocyte subsets, after toxin pretreatments. Cells were preincubated with 10, 100, or 1000 ng/mL LT, ET, or LT+ET for 3 h, followed by 120 min of spore exposure at 10 spores/cell. Cytochalasin D (5 μM) pretreatment for 1 h prior to spore addition served as a control for internalization inhibition. Graphs show percent change in phagocytic indexes (PIs) compared to no treatment. PIs were calculated as the percent of a given cell subset positive for pHrodo signal multiplied by the median fluorescence intensity of the pHrodo-positive cells of that subset. (**A**) % change in PIs of RAW 264.7 cells. Graphs represent mean + SEM with results from 3 independent experiments overlaid (green). (**B**) % change in PIs of human AARP subsets. Graphs represent mean + SEM with individual results from 7 lung donors overlaid (blue). (**C**) % change in PIs of human leukocyte subsets. Graphs represent mean + SEM with individual results from 5 blood donors overlaid (red). NS = not significant, * *p* ≤ 0.05, ** *p* ≤ 0.01, *** *p* ≤ 0.001, **** *p* ≤ 0.0001, Friedman test with Dunn’s multiple comparisons test to no treatment. Black arrows indicate trends based on *p* values less than 0.20.

We finally tested if anthrax toxins altered cellular function by measuring *B. anthracis* spore internalization in the different cell types (Figure 5 and Figure 6). We previously showed that all six human AARP subsets internalize anthrax spores within 30 min of exposure [10]. However, we also determined that human alveolar epithelial cells internalize spores [52], providing a transcellular passageway for spores to the lymphatic system and ultimately the mLN. Therefore, we chose spores as a relevant marker for phagocytosis in AARPs, and as general marker for phagocytosis and/or non-phagocytic particle internalization in leukocytes. For all internalization assays, we began by preincubating cells for 3 h with increasing concentrations of LT, ET, or a combination of LT and ET. Cells were pretreated with 5 μM cytochalasin D (an inhibitor of actin polymerization) as a positive control for inhibition of phagocytosis. We then exposed the cells to pHrodo-labeled *B. anthracis* spores at ~10 spores/cell for 30 min to represent early spore interactions (Figure 5) or 120 min to represent late spore interactions (Figure 6). pHrodo is a pH-sensitive dye that fluoresces upon acidification [10,53,54], as occurs in phagocytes after spore uptake. The dye is nonfluorescent at neutral pH, as occurs outside the cell. As spores are internalized and subsequently acidified within phagosomes, pHrodo fluorescence dramatically increases. To measure spore internalization, we first calculated a flow cytometric phagocytic index (PI), as we have done previously to measure pathogen internalization [10]. This value was calculated as the percentage of the cell population positive for pHrodo signal multiplied by the pHrodo median fluorescence intensity of pHrodo-positive cells. We then plotted the PIs as percentages of the untreated control. As expected, untreated RAW 264.7 rapidly internalized *B. anthracis* spores (within 30 min), and internalization was significantly diminished by cytochalasin D treatment (Figure 5A). Additionally, LT at 1000 ng/mL, as well as LT/ET at 1000 ng/mL, significantly decreased early spore internalization by RAW 264.7 cells (Figure 5A). ET alone at concentrations as high as 1000 ng/mL had no effect on spore uptake. As we already determined that LT induced apoptosis in these cells (Figure 3A), we suspected that decreased spore internalization was linked to this apoptotic effect. We then performed the same treatments on human AARPs, to determine if LT or ET inhibited early internalization in these cells (Figure 5B). Again, cytochalasin D pretreatment inhibited spore internalization in all the AARP subsets. Interestingly, early internalization was significantly decreased in AM, as well as in BDCA1− CD14−, BDCA1+ CD14+, and BDCA1+ CD14− cells pretreated with the combination of LT and ET at concentrations of 100 and/or 1000 ng/mL (Figure 5B). LT or ET alone at these concentrations did not significantly decrease early internalization, although there were trends (*p* < 0.20) suggesting ET was driving this effect in AM and both toxins were contributing to this effect in BDCA1− CD14− cells (Figure 5B). We then tested the effect of LT and ET on early spore internalization by human leukocytes (Figure 5C). Spore uptake by BDCA1+ CD14− DCs was significantly decreased by the combination of LT and ET at 1000 ng/mL. B cells, granulocytes, and monocytes also showed trends of inhibition caused by combined LT and ET at 1000 ng/mL. LT was likely causing this effect based on inhibition trends observed with LT-only exposure at 1000 ng/mL in BDCA1+ CD14− DCs and monocytes (Figure 5C). ET alone had no effect on spore internalization by human leukocytes. Thus, anthrax toxins decreased early spore internalization in several AARP and leukocyte subsets 

We next tested whether LT or ET decreased late spore internalization in AARPs and leukocytes. We accomplished this by preincubating cells with toxins for 3 h, followed by 120 min of spore exposure (Figure 6). The LT-induced inhibition of spore internalization was still evident in RAW 264.7 after 120 min of spore exposure (Figure 6A). LT at 1000 ng/mL, as well as LT plus ET at 1000 ng/mL, decreased spore internalization compared to untreated cells. Several AARP subsets showed significantly decreased late spore internalization due to toxin exposure (Figure 6B). Surprisingly, this effect was largely due to ET exposure and not due to LT. Specifically, spore internalization by AM, BDCA1− CD14+ cells, and Langerin+ cells was significantly decreased by ET exposure alone at 1000 ng/mL. The other three AARP subsets showed trends of decreased internalization at 1000 ng/mL ET. None of the AARP subsets displayed significantly decreased internalization after LT exposure alone, although the three members of the MΦ/monocyte clade did show trends of inhibition at 1000 ng/mL. The BDCA1− CD14− subset was the only AARP in which LT and ET together significantly decreased spore internalization. In this subset, LT or ET alone at 1000 ng/mL caused trends of decreased internalization, but only the combination of LT and ET at 1000 ng/mL resulted in statistically significant inhibition of internalization. Late spore internalization was also decreased in all the leukocytes after toxin exposure (Figure 6C). In contrast to the AARPs results, this effect was clearly driven by LT exposure, and not by ET exposure. In all seven leukocyte subsets, LT alone at 1000 ng/mL significantly decreased internalization. However, ET did not have any effect on internalization at concentrations as high as 1000 ng/mL. LT and ET in combination at 1000 ng/mL decreased internalization in all leukocytes. Thus, these results suggest that to some extent early but especially late spore internalization by human AARPs and leukocytes is inhibited by anthrax toxins. ET is primarily responsible for the decreased internalization measured in AARPs, although LT likely contributes to this effect to a lesser degree. In contrast, peripheral blood leukocyte spore internalization is specifically inhibited by LT exposure and is not affected by ET. This decreased spore internalization as a result of LT was evident in phagocytic (granulocytes, BDCA1+ CD14− DCs, and monocytes) and non-phagocytic (B cells, NK cells, NKT cells, and T cells) cells, suggesting LT targets global particle internalization pathways.

## 3. Discussion

Our study examined the effects of anthrax LT and ET on human phagocytic subsets in the lung. Further, the results systemically compare these effects between the RAW 264.7 mouse MΦ cell line commonly used in anthrax studies [37,41,44,55,56,57,58], six AARP subsets we have previously characterized [10], and seven subsets of human leukocytes. We demonstrate that: (1) The majority of resting AARPs and leukocytes do not express surface TEM8 and CMG2 based on flow cytometric analysis (Figure 1); (2) all the AARPs and leukocytes bind/internalize anthrax PA in a dose-dependent but toxin receptor-independent manner (Figure 2); (3) AARPs and leukocytes are resistant to LT-induced apoptosis (Figure 3) and necrosis (Figure 4) at toxin concentrations as high as 1000 ng/mL and exposure time as long as 3 h; (4) early spore internalization by some AARPs and leukocytes is inhibited by anthrax toxins (Figure 5); and (5) late spore internalization by AARPs is inhibited by ET exposure, while internalization by leukocytes is inhibited by LT exposure (Figure 6). We discuss these findings individually below.

Considering RAW 264.7 cells are known to be sensitive to LT-induced lethality [37], it was interesting that only 30% expressed surface TEM8 and less than 5% expressed surface CMG2 (Figure 1A). This is in contrast with previous work suggesting TEM8 was not detectable on RAW 264.7 cells, while about 40% of these cells expressed surface CMG2 [42]. Other studies have examined receptor expression at the mRNA level or in cell lysates. We previously showed that CMG2 transcript levels are higher than TEM8 levels in RAW 264.7 cells [41]. However, we were able to measure TEM8 protein in cell lysates but were unable to verify CMG2 protein in the same lysates. Premanandan et al. [43] determined that RAW 264.7 cells expressed both TEM8 and CMG2 transcripts, while Banks et al. [44] could only measure CMG2 mRNA. These discrepancies at the transcript and protein levels may relate to differing culture conditions. Freshly cultured RAW 264.7 cells may express higher levels of CMG2 and lower levels of TEM8. Our measurements were conducted on RAW 264.7 cells that had been grown to near confluency prior to harvest for flow cytometry. Some of the differences in surface staining may relate to differences in the specific antibodies used. The rarity of many of the AARP and leukocyte populations examined in this work precludes confirmatory analysis by standard Western blotting or RT-PCR. We do acknowledge that more sensitive techniques than flow cytometry, such as single-cell Westerns and single-cell RNAseq, may have yielded different expression results. However, our current and past work using transcript measurement, immunoblotting, and flow cytometry is likely to be more reliable than studies using single detection techniques. 

Out of the six AARP subsets we investigated, none showed more than 20% positivity of either TEM8 or CMG2 by flow cytometric analysis. The low expression of TEM8 and negligible expression of CMG2 on AMs (Figure 1B) are consistent with our previous findings that showed that AMs expressed low levels of TEM8 protein and no detectable levels of CMG2 by immunoblotting analysis [41]. The physiological roles of TEM8 and CMG2 remain unknown, although they have been linked to angiogenesis, cell migration, and extracellular matrix remodeling [59,60,61]. It is not surprising that receptor positivity was low on the AARP subsets, as both TEM8 and CMG2 were first identified on and are expressed highly by endothelial cells [60,62]. Aside from PA, TEM8 has an affinity for collagen I, collagen VI, and gelatin [63,64,65], while CMG2 binds to collagen IV, fibronectin, and laminin [62]. All these ligands are commonly found along endothelial and connective tissues, especially as part of the extracellular matrixes. Another surface receptor, integrin β1, has been shown to bind PA as well, and may function as a third receptor for PA due to a shared von Willebrand factor A component with TEM8 and CMG2 [66]. We did not measure the levels of this integrin on human AARPs or leukocytes. Liu et al. showed that in mice, double knockouts of TEM8 and CMG2 are resistant to both toxin-induced lethality and subcutaneous spore challenge [31], suggesting that even if a third receptor exists, it is not relevant to in vivo pathogenesis. This group also demonstrated that TEM8 knockout mice were still susceptible to anthrax toxin and spore challenges, while CMG2 knockouts were resistant to both [31,67]. Therefore, at least in a rodent model, CMG2 expression is the predominant cellular receptor allowing toxin-induced effects. However, it is unknown whether this is true for inhalation anthrax in humans. 

For the analysis of human leukocytes, we followed the cell identification strategy outlined by Ingram et al [46], with the addition of granulocytes and BDCA1+ CD14− DCs (Figure 1C). We chose to include granulocytes, as neutrophils, which make up the majority of this category, are known to be affected by anthrax toxins at the level of phagocytosis [68]. Additionally, mice depleted of neutrophils are susceptible to infection after subcutaneous spore challenge [69]. We acknowledge that eosinophils and basophils would also be included in our granulocyte grouping, and could show different toxin susceptibilities compared to neutrophils. However, eosinophils and basophils are a very small fraction of leukocytes from healthy individuals [70,71,72]. Similarly, we did not separate the other leukocyte categories into additional subsets, such as dividing T cells into T helper cells and cytotoxic T cells. In future studies, we may expand our analyses of leukocyte subsets based on our current results. The seven leukocyte populations we investigated also did not show high positivity for TEM8 and CMG2 (Figure 1D). Granulocytes, monocytes, and BDCA1+ CD14− DCs showed the highest TEM8 positivity, but none of these levels were greater than 15%. The non-phagocytic NK cells, NKT cells, T cells, and B cells all displayed less than 5% positivity. CMG2 surface expression was highest in B cells, but even this level was just above 5%. It is possible that time in culture could lead to increased surface expression, as the cells we examined were stained and analyzed freshly after thawing. Interestingly, prior exposure to anthrax toxins in the form of anthrax vaccination or recovery from cutaneous infection appears to modulate future TEM8 expression. Ingram et al. [46] showed that human monocytes from naïve individuals have either high or low TEM8 expression. It is possible that had we sampled blood from more individuals, we would have seen more spread in the TEM8 and CMG2 surface expression. Vaccinated individuals have been shown to have TEM8-high-expressing monocytes, while convalescent individuals all had TEM8-low-expressing monocytes [46]. Our results agree with this group’s work in that surface expression of TEM8 on NKT cells, T cells, and NK cells is quite low. However, they measured upwards of 70% positivity on B cells, while we only measured 6% on our cells. We do not know the anthrax vaccination or infection history of our blood donors, but in the United States, they are much less likely to fit either category. Other vaccinations or infections could also affect the expression of TEM8 and CMG2. We would expect both to be different, as the naïve blood donors in the other study were from the United Kingdom [46]. Interestingly, ET exposure for 24 h upregulates TEM8 and CMG2 RNA levels in RAW 264.7 cells [73]. It remains unknown whether a similar effect occurs in primary human cells. In mice, CMG2 is the major toxin receptor conferring lethality to anthrax infection [67,69]. CMG2 expression in human lymphocytes is genetically (rather than environmentally) determined, and CMG2 mRNA levels strongly correlate (11–24%) with cellular toxin susceptibility [32]. However, these results also suggest that 76–89% of the variability in toxin sensitivity is not accounted for by CMG2 expression. The range of relative toxin susceptibility differed by four orders of magnitude within lymphocytes from a cohort of 234 people. Thus, toxin receptor expression is highly variable in humans as a result of genetic and environmental factors, as well as the cell type analyzed. Our data suggests than unstimulated human AARP subsets do not express high levels of surface TEM8 or CMG2.

However, receptor expression alone does not necessarily correlate with PA binding and toxin internalization in human cells. Ingram et al. [46] determined that less than 20% of NK, NKT, and T cells expressed TEM8, yet 55% to 65% of these cells bound dye-labeled PA. For this reason, we also measured PA binding/internalization by RAW 264.7 cells, human AARP subsets, and human leukocyte subsets (Figure 2). It is known that after PA binds to TEM8 or CMG2, LT and ET are internalized via actin-dependent clathrin-mediated endocytosis [33]. From there, acidification of the endosome leads to formation of a pore in the endosomal membrane, detachment of LF and EF from PA, and the release of both components into the cytosol, leading to cellular effects [34,74,75]. However, we propose that anthrax toxins can enter cells via non-specific receptor-independent mechanisms as well. As expected, PA bound to RAW 264.7 cells in a dose-dependent manner from 10 to 1000 ng/mL (Figure 2A). When we preincubated these cells with unlabeled PA at 3X the concentration of the labeled PA, it did significantly decrease binding. However, additional preincubation at 10X unlabeled did not further decrease PA binding, suggesting a surface receptor-independent mechanism of PA binding/internalization. Phagocytic cells, such as MΦ and DCs, constitutively sample the extracellular environment and internalize soluble molecules by a receptor-independent process called macropinocytosis [76,77,78]. The resulting vesicular compartments, identified as macropinosomes, eventually fuse with lysosomes and undergo acidification [79]. Our analysis of labeled PA binding by flow cytometry would include surface-bound PA to TEM8 and CMG2, as well as PA internalized by macropinocytosis. This non-specific mechanism of internalization explains why 10,000 ng/mL unlabeled PA did not further decrease labeled-PA binding, as liquid-phase sampling by macropinocytosis is not saturable. 

Like the RAW 264.7 cells, all the AARP subsets, including AM, displayed dose-dependent increases in PA binding (Figure 2B). However, only Langerin+ cells showed a significant decrease in binding when preincubated with 3X unlabeled PA. This suggests that most of the PA signal we were measuring in all the AARP subsets was not from surface interactions with TEM8 and CMG2 but rather by PA internalization via macropinocytosis. We acknowledge that in a previous study, we detected very low levels of PA binding by human AMs [41]. AMs are known to be highly autofluorescent, and this autofluorescence can be used as part of their identification schema [10,80]. In our earlier work [41], we utilized PA labeled with fluorescein isothiocyanate (FITC) dye, which is detected in the same wavelength range as the autofluorescence of AM. Thus, autofluorescence was likely masking the true FITC-PA signal [81]. In our current study, we labeled PA with AF647, which has an emission spectrum that is unaffected by the inherent autofluorescence in AM. In the analysis of PA binding by leukocytes, all seven populations bound/internalized PA in a dose-dependent manner (Figure 2C). Four of the subsets showed significantly decreased PA binding when preincubated with 3000 ng/mL unlabeled PA, but none of the seven subsets showed additional inhibition of binding when incubated with 10,000 ng/mL unlabeled PA. Therefore, B cells, BDCA1+ CD14- DCs, monocytes, and NKT cells do express physiologically active surface TEM8 and CMG2. However, the majority of PA binding that we measured was likely due to macropinocytosis. Recent studies have demonstrated that non-professional phagocytes, such as B cells and T cells, also perform macropinocytosis [82,83]. Therefore, it is likely that NK cells and NKT cells can also sample their environment via this internalization mechanism. Additionally, macropinocytosis is a very rapid process, with the entire cell membrane of phagocytes being internalized within 30 min while sampling the extracellular environment [84]. Further supporting the involvement of macropinocytosis, professional phagocytes, which perform constitutive macropinocytosis [76,77,78,85], bound/internalized far more labeled PA than non-phagocytes (Figure S1). After verifying PA was internalized by AARPs and leukocytes, we wanted to determine if the toxins affected the viability of these cell types.

For some time now it has been known that anthrax LT induces apoptosis in susceptible cell types, such as RAW 264.7 murine MΦ [37,41]. We also measured a significant induction of apoptosis in RAW 264.7 cells as a result of LT exposure at 1000 ng/mL (Figure 3A). Interestingly, while ET alone caused no apoptosis of RAW 264.7 cells, LT in combination with ET significantly increased apoptosis at concentrations ranging from 10 to 1000 ng/mL. This suggests that ET can act with LT to cause global cellular toxicity in RAW 264.7 cells. A previous work showed that ET does not directly induce RAW 264.7 cell death [86]. However, ET treatment does dramatically increase intracellular cAMP levels in these cells and subsequently triggers cell cycle arrest [87]. Additionally, ET alone does upregulate a number of apoptosis regulators at the transcriptional level [86]. Thus, it is plausible that ET sensitizes RAW 264.7 cells to the induction of apoptosis by lower concentrations of LT. 

These results led us to determine if any of the rare AARP subsets we recently characterized [10] were sensitive to LT-induced apoptosis in the presence or absence of ET. Our group previously demonstrated that human AMs are resistant to LT-induced apoptosis over 3 h of toxin exposure [41], and we verified this finding in the current study, despite the differences we found in PA binding as discussed earlier. However, we did not previously test their sensitivity with ET exposure included. If any of the AARP subsets showed sensitivity to LT-induced apoptosis, they could be implicated as targets of *B. anthracis* during early stages of inhalation anthrax. Yet, LT, ET, or LT with ET did not trigger apoptosis in any of the AARP subsets, at least at concentrations as high as 1000 ng/mL and for times as long as 3 h (Figure 3B). Therefore, we showed that if LT and/or ET were affecting human AARP subsets, it was not via direct rapid induction of apoptosis. We also did not find toxin-mediated induction of apoptosis in any of the seven leukocyte subsets tested under the same specific conditions (Figure 3C). Others have shown that LT treatment induces apoptosis in human monocytes [88]. However, these treatments were conducted under different conditions than our studies. As monocytes undergo spontaneous apoptosis in culture without stimulation [89,90], Popov et al. [88] pretreated the monocytes with interferon gamma (IFN-γ) for 48 h to extend their survival. Additionally, their LT exposures were over a length of 15–24 h after activation. Human monocyte-derived DCs also undergo LT-induced apoptosis, although this effect occurs after 48 h of LT exposure [50]. Our current study examined the immediate response of human AARPs and leukocytes to anthrax toxins after 3 h of exposure. We specifically wanted to measure the early effects of anthrax toxins on unstimulated cells. Additionally, we observed that AARP viability decreases between four and eight hours in culture [10], preventing experiments with exposures for longer periods of time. Our AARP results agree with our previous findings that demonstrated that human AM are resistant to LT-induced apoptosis after 3 h of exposure [41]. We acknowledge that higher toxin concentrations (> 1000 ng/mL) or longer exposure times may have revealed the sensitivity of some of the AARP and/or leukocyte subsets to LT-induced apoptosis. Human neutrophils are also resistant to toxin-induced apoptosis, even though they experience LT-induced MEK cleavage and ET-induced cAMP surges [91]. In fact, aside from activated monocytes, the only other primary human cell type shown to undergo LT-induced apoptosis have been endothelial cells [92]. 

However, apoptosis is not the only type of cell death known to occur in response to anthrax toxin exposure. Bone marrow-derived MΦ from certain mouse strains and human monocyte-derived MΦ have been shown to undergo caspase 1-mediated necrosis within 2 h in response to LT [50,51]. We therefore also measured necrosis in RAW 264.7 cells, human AARPs, and human leukocytes (Figure 4). In RAW 264.7 cells, LT induced necrosis at 1000 ng/mL, while ET induced necrosis at 100 and 1000 ng/ mL (Figure 4A). These results suggest that LT and ET both induce necrosis in RAW 264.7 cells, and further that these cells are more sensitive to the ET effect than the LT effect. However, the combination of LT and ET did not cause necrosis at any of the tested concentrations. As LT plus ET induced apoptosis in RAW 264.7 cells at all tested concentrations (Figure 3A), it is possible that mechanistically, the toxins together can induce apoptosis but not necrosis. Park et al. demonstrated that ET promotes RAW 264.7 survival after LT exposure through the transcription factor cAMP response element-binding protein (CREB) [93]. Our results did show that the level of apoptosis induced by LT plus ET was less than LT alone (Figure 4A). Similarly, LT may act to inhibit the induction of necrosis when the two toxins are together. All the AARP subsets (Figure 4B) and leukocytes (Figure 4C) tested were resistant to LT-induced necrosis at concentrations up to 1000 ng/mL and exposure times up to 3 h. Additionally, ET did not trigger necrosis in these cells by itself or in combination with LT. These results are not surprising, as to our knowledge, ET has not been previously associated with necrosis in primary human cells. Thus, at least at early times, human AARPs and leukocytes are resistant to the known effects of LT-induced necrosis. As with the induction of apoptosis, it is possible that higher toxin concentrations or longer exposure times could have caused necrosis in some of the AARP and/or leukocyte populations. We should note that we unexpectedly observed a significant decrease in necrosis as a result of PA exposure alone in four of the leukocyte populations (Figure 4C). While the physiological importance of this finding, if any, is unknown at this time, PA alone has been shown to decrease cell growth and metabolic activity in CHO cells expressing human TEM8 and CMG2 [94]. This may affect spontaneous necrosis of these cells.

After not observing induction of apoptosis or necrosis in human AARPs or leukocytes, we decided to examine whether early (30 min) and late (120 min) *B. anthracis* spore internalization was affected by LT and/or ET exposure (Figure 5 and Figure 6). Others have shown that RAW 264.7 cells exposed to LT at concentrations as low as 8 ng/mL display decreased phagocytosis of *B. anthracis* spores [37]. However, this group did not perform spore exposure in non-germinating conditions, incubated with LT for a longer period of time, and used Alamar Blue visualization to quantify spores; making direct comparisons to our results impractical. We used percent changes instead of direct phagocytic indexes so as to allow comparison between cells of different phagocytic capacity, and also between non-phagocytic leukocytes. In our current experiments, RAW 264.7 cells displayed decreased early spore phagocytosis by 30 min of LT exposure at 1000 ng/mL. This effect was only due to LT exposure, as ET alone showed no effect, while LT plus ET at 1000 ng/mL caused the same inhibition (Figure 5A). As suggested by Popov et al. [37], it is likely that induction of apoptosis by LT in these cells (Figure 3A) contributes to decreased phagocytosis. This decreased internalization continued to occur through late spore exposure (Figure 6A), suggesting long-term phagocytic impairment of these cells. 

We previously showed that human AARP subsets display significantly different phagocytic potential of *B. anthracis* spores [10]. Intriguingly, human AARPs display decreased early and late spore phagocytosis in response to toxin preexposure (Figure 5B and Figure 6B). In AM, this inhibition is driven by ET, while LT has no effect. These results are consistent with our previous findings that show human AMs are resistant to LT-induced MEK cleavage and apoptosis [41]. Others have shown that *B. anthracis* spores and vegetative bacilli stimulate the secretion of an antibacterial phospholipase, Group II secretory phospholipase A2 (sPLA2-IIA), by guinea pig AMs [95]. However, ET specifically inhibits this production of sPLA2-IIA via a cAMP-dependent pathway. Therefore, multiple innate immune functions of AM are affected by ET. The rarer AARP subsets also showed decreased spore phagocytosis at early and late exposures. At early spore exposures, we mainly observed inhibition from the combination of LT and ET treatments at 100 and 1000 ng/mL, while LT or ET alone did not significantly decrease spore uptake (Figure 5B). This suggests that the toxins work in combination to inhibit early phagocytosis in the rarer AARPs. However, at late spore exposure, we measured the inhibition was primarily through ET exposure, with LT having less influence on spore internalization (Figure 6B). Cyclic AMP levels are known to transiently increase during phagocytosis by MΦ [96] and neutrophils [97,98]. Interestingly, cAMP does not freely diffuse throughout the cell but is rather compartmentalized to the phagosome, while remaining low in the cytosol [99]. Elevation of cAMP is known to inhibit phagocytosis by MΦ [100,101], but more specifically, this is likely increased cytosolic cAMP. Finally, after internalization and acidification, PA is known to form a pore through which EF escapes into the cytosol to exert its cellular effects [21]. In the cytosol, increased cAMP levels caused by EF would disrupt the cAMP gradient that exists between the maturing phagosome and the cytosol, resulting in overall decreased phagocytosis. Our AARP results collectively provide a model of early stage inhalation anthrax, in which some *B. anthracis* spores germinate in the alveoli and begin producing toxin components. LT and ET enter the six human AARP subsets via mainly macropinocytosis (Figure 1 and Figure 2). Human AARPs are resistant to LT-induced apoptosis and necrosis (Figure 3 and Figure 4), yet they are sensitive to ET-induced inhibition of phagocytosis (Figure 5 and Figure 6). Spores and bacilli would normally be phagocytosed and killed by these AARPs within the alveoli, but with decreased internalization by these sentinel cells, the pathogen can cross the alveolar epithelium by transcellular [16,18,52] and/or paracellular [19] routes to transit via the lymphatics to the mLN. In this model, it remains unclear how toxins are escaping from macropinosomes to exert their effects despite minimal TEM8 or CMG2 expression. Acidification of macropinosomes occurs by lysosomal fusion [77,102], which would allow separation of LF and EF from PA. Others have shown that ET exposure causes upregulation of TEM8 and CMG2 in phagocytes [73,103], so it is possible that receptor upregulation occurs prior to degradation of the contents of the macropinolysosome, allowing LF and EF escape to the cytosol. Localization of toxin components in AARPs will be a focus of future studies by our group.

In the peripheral blood leukocyte populations, *B. anthracis* late spore internalization was inhibited in both phagocytic and non-phagocytic cells by anthrax toxin exposure (Figure 5C and Figure 6C). Surprisingly, and in contrast to our AARP results, the inhibition of spore internalization was predominantly caused by LT and not by ET exposure. Few studies have examined internalization of particles by human leukocytes after anthrax toxin exposure. While leukocytes are not exposed to spores during the course of inhalation anthrax pathogenesis [6,7,104], we used them in our studies for comparative purposes. We predict that the LT-induced inhibition of internalization in leukocytes is actually a global effect not specific to spores. During the later stages of inhalation anthrax, vegetative bacilli rapidly multiply in the bloodstream, reaching concentrations as high as 10^8^ bacilli/mL blood [1]. Phagocytic cells in the blood, such as neutrophils, monocytes, and BDCA1+ CD14− DCs, would serve to defend the bloodstream during early bacteremia to prevent the development of septicemia. The downregulation of internalization we observed in non-phagocytic cells may be a bystander effect of LT exposure, whereas other cellular functions are more importantly downregulated in these cells. For example, LT exposure decreases T cell receptor signaling and associated cytokine production in NKT cells [105]. Additionally, B cells experience decreased antibody production and proliferation as a result of LT exposure [106]. Relating to phagocytes, previous work has shown that human neutrophils exhibit decreased phagocytosis of opsonized, irradiated, and vegetative *B. anthracis* after preincubation with ET but not with LT [68]. It is possible that ET affects phagocytosis of opsonized bacilli, while LT affects that of non-opsonized spores. Additionally, this group exposed neutrophils to bacilli at a 30 cfu:1 cell ratio and only preincubated with toxins for 1 h. We used a 10 spores:1 cell ratio and preincubated with toxins for 3 h. Thus, the phagocytic inhibition of neutrophils by ET [68] may be apparent at high particle exposures and transient. By 3 h of toxin exposure, the effects of ET on internalization by neutrophils had diminished. Based on our findings, if ET does affect internalization by leukocytes, it is through a different pathway than in AARPs. While LT does not cause apoptosis or necrosis of human neutrophils, it inhibits actin-based motility within 2 h of exposure [107]. LT also cleaves MEKs and affects actin regulation at the transcriptional level in human monocytes [108]. Phagocytosis is an actin-dependent process [109,110,111]. Thus, it is likely that MEK cleavage caused by LT affects actin cytoskeletal arrangement and thus suppresses phagocytosis in leukocytes like neutrophils and monocytes. Overall, our studies suggest that ET may play a role in early stages of inhalation anthrax by preventing spore phagocytosis, and thus intracellular killing, by AARPs, which allows alveolar escape. In late-stage disease, LT assumes the major role by inhibiting phagocytosis of vegetative bacilli in the bloodstream, which impairs bacterial clearance and allows rapid replication that overpowers innate immune defenses. 

Future studies will continue to investigate the effects of LT and ET on human AARPs and leukocytes. A primary aim will be to further determine the mechanism(s) by which LT and ET enter human AARPs and leukocytes to exert their cellular effects. These studies will involve the tracking of toxin components to allow compartmental localization within the different cell types. This work will allow us to identify the extent to which macropinocytosis is involved in toxin uptake, and further, how LT and ET can escape macropinosomes. We will also examine the effects the toxins have on spore survival within the phagocytes of the lung and blood. While our previous studies, and those of others, have used LT and ET added extracellularly, it is also possible that toxin produced intracellularly by germinating spores can affect phagocyte functions. These effects could be at the levels of phagosome maturation, cytokine production, antigen presentation, motility, or a number of other functions commonly associated with phagocytes. Lastly, we will perform transcriptional profiling of the AARP subsets after toxin exposure. We have already compared the transcriptomes of the six AARP subsets under resting conditions [10], and thus it would be beneficial to the field to identify genes that are up- or downregulated upon LT and/or ET exposure. 

## 4. Materials and Methods 

### 4.1. Collection of Human Bronchoalveolar Lavage (BAL) Cells

Primary human AARPs were collected by lavage of whole donor lungs and preserved as previously described [10]. Assay parameters and methodologies were developed using cells from donor lungs obtained through LifeShare of Oklahoma (Oklahoma City, OK; USA; http://www.lifeshareoklahoma.org), with donor criteria described below. Cells from the same seven lung donors used in our previous AARP classification and characterization study [10] were used for acquiring data presented in the current study. Briefly, human donor lungs were obtained through the International Institute for the Advancement of Medicine (Edison, NJ, USA; http://www.iiam.org), a nonprofit division of MTF Biologics. Lungs were healthy but deemed nontransplantable for reasons, such as histocompatibility mismatching, size discrepancies, uncertain drug usage, or prior incarceration. We accepted lungs based on a strict set of criteria, including: 18–70 years of age, nonsmoking a minimum of 2 years, no history of lung disease, noncardiac death, a PaO_2_/FiO_2_ ratio > 200, and normal to minimal atelectasis based on chest X-ray results, with no evidence of intercurrent infection. The donor demographics for the seven lungs used in this study can be found in Supplementary Material Table S1 of our previous work [10]. Upon arrival, Wisconsin solution and residual blood were washed from the vasculature using sterile physiological saline (0.9% w/v). Saline was pumped at low pressure (~20 cm H_2_O) into the main bronchus to produce visible swelling of lobes. The resultant bronchoalveolar collections were pooled, and cells were concentrated by centrifugation at 300× *g* for 10 min, resuspended to 1 × 10^7^ cells/mL in freeze medium (40% RPMI 1640, 50% fetal bovine serum (FBS), and 10% dimethyl sulfoxide (DMSO)) [112], frozen at a rate of ~1 °C/min at −80 °C, and stored in liquid nitrogen vapor at −190 °C.

### 4.2. Collection of Human Leukocytes

Freshly collected buffy coats were provided by the Oklahoma Blood Institute upon request. All five blood donors for leukocyte experiments described in this study were obtained in this manner. Blood donors (2 men and 3 women) were in good health and tested negative for all infections screened for during standard blood donation. Age and smoking history of blood donors were not available. Leukocytes were immediately isolated by gradient centrifugation using Lymphoprep (Stemcell Technologies; Vancouver, BC, Canada) according to the manufacturer’s instructions. Leukocytes fractions were pooled, concentrated by centrifugation, and preserved in freeze medium using the same reagents and techniques as described for human BAL cells. 

### 4.3. Flow Cytometry

All flow cytometric data for RAW 264.7 cells, human AARPs, and human leukocytes were acquired on a BD LSR II (BD Biosciences; San Jose, CA, USA). Data were analyzed using FlowJo v10 software. All cells were pretreated with blocking antibodies to mouse (RAW 264.7 cells) or human Fc receptors prior to primary antibody incubation. Monoclonal/polyclonal antibodies and viability dyes used for all experiments are listed in Table S1. Cell gates were determined using fluorescence minus one controls for each stain, with the minus stain being filled with a labeled isotype control to account for nonspecific binding.

### 4.4. Preparation of Bacillus anthracis Spores

*B. anthracis* spores were specifically used for internalization assays after toxin exposure. A starter culture of germination-deficient *B. anthracis* (Sterne strain 34F2 Δ*ger*) was provided by the laboratory of Dr. Philip Hanna (University of Michigan, Ann Arbor, MI, USA) [113]. Spore stocks were created as previously described [114]. Briefly, bacteria were grown with continuous shaking in Luria–Bertani medium overnight at 37 °C. On the next day, bacteria were streaked on AK agar sporulating dishes and incubated for 3 weeks at 30 °C. At the time of harvest, each dish was washed by pipetting with 5 mL of chilled, sterile, and deionized water to dislodge spores. Spore collections were spun at 10,000× *g* for 10 min and resuspended in 1 mL of chilled water. Spore suspensions were heated at 65 °C for 60 min to kill any vegetative bacteria. After heat treatment, spores were centrifuged for 10 min at 10,000× *g*, and the supernatant was discarded. Pellets were resuspended in 1 mL of chilled water and centrifuged for 10 min at 10,000× *g*. The upper layer of the resulting spore pellets was washed by pipette to remove contaminating bacterial cell debris. The supernatant and the top layer of the pellet were aspirated and discarded. The wash procedure was repeated a total of five times, with the final spore preparations being resuspended in chilled, sterile, and deionized water, and pooled. Spore counts were determined by a hemocytometer and stocks were stored at 4 °C. All spores in experiments were harvested < 30 days prior to use.

### 4.5. Anthrax Receptor Surface Expression

The mouse macrophage cell line RAW 264.7 (ATCC^®^ TIB-71™; Manassas, VA, USA) was cultured in RPMI 1640 with 10% heat-inactivated fetal bovine serum (Millipore Sigma, USA origin; RPMI-10) and split when near 80% confluence. On the day of assays, cells were harvested from culture flasks with Accutase Cell Detachment Solution, centrifuged at 300× *g* for 10 min, and resuspended at 1 × 10^6^ cells/100 μL in 1X PBS. Cells were then stained with Zombie Aqua fixable viability dye (BioLegend; San Diego, CA, USA) on ice following the manufacturer’s instructions, followed by 10 min FcR blocking on ice with Mouse TruStain FcX (anti-mouse CD16/32) antibody (BioLegend; San Diego, CA, USA). RAW 264.7 cells were then incubated with primary rabbit antibodies against mouse TEM8 and CMG2 (Table S1) for 20 min on ice. Finally, cells were stained with phycoerythrin (PE) -conjugated donkey anti-rabbit antibodies. All samples were centrifuged at 300× *g* for 5 min and washed twice with 1X PBS-2. Samples were fixed with 2% paraformaldehyde prior to flow cytometry. 

For human BAL cells and blood cells, preserved samples were rapidly thawed at 37 °C, resuspended in RPMI-10, and centrifuged at 300× *g* for 10 min. Supernatants were discarded and the cell pellets depleted of dead/dying cells using a dead cell removal kit (Miltenyi Biotec; Bergisch Gladbach, Germany) in conjunction with MACS MS separation columns (Miltenyi Biotec; Bergisch Gladbach, Germany) according to the manufacturer’s instructions. Total viable cell counts were determined by trypan blue exclusion and cells were resuspended at 1 × 10^6^ cells/100 μL in 1X PBS. Cells were then stained with Zombie Aqua fixable viability dye (BioLegend; San Diego, CA, USA) on ice following the manufacturer’s instructions, followed by 10 min of FcR blocking on ice with Human TruStain FcX blocking solution (BioLegend; San Diego, CA, USA). Finally, human lung and blood cells were stained with fluorescently labeled Abs against TEM8, CMG2, and the necessary cell identification markers (Table S1), following the gating strategies outlined previously [10] and in this study (Figure 1C), for 20 min on ice. All samples were centrifuged at 300× *g* for 5 min and washed twice with 1X PBS-2. Samples were fixed with 2% paraformaldehyde prior to flow cytometry. 

### 4.6. Protective Antigen Binding Assays

For all PA binding assays, activated PA (63 kDa, List Biological Laboratories, Inc.; Campbell, CA, USA) was labeled using an Alexa Fluor® 647 Microscale Protein Labeling Kit (Molecular Probes; Waltham, MA, USA) following the manufacturer’s instructions. RAW 264.7, human BAL cells, and human leukocytes were processed as described for anthrax surface receptor expression through the point of Fc receptor blocking. At this point, cells were either left untreated, or incubated with 3000 or 10,000 ng/mL unlabeled PA as indicated for 30 min on ice. Cells were then exposed to AF647-labeled PA at 10, 100, or 1000 ng/mL for an additional 30 min on ice. In the case of lung and blood cells, antibodies used for cell subset-specific identification were also added at the same time as AF647-PA. All samples were centrifuged at 300× *g* for 5 min and washed twice with 1X PBS-2. Samples were fixed with 2% paraformaldehyde prior to flow cytometry. 

### 4.7. Apoptosis and Necrosis Induction

Specific toxin components used in exposures were PA (83 kDa), LF (native sequence), and/or EF (all components from List Biologicals Laboratories, Inc.; Campbell, CA, USA). Cultured RAW 264.7 cells were harvested with Accutase, quantified, centrifuged at 300× *g* for 10 min, and resuspended in RPMI-10 containing 100 IU/mL penicillin, 100 mg/mL streptomycin, and 2.5 mg/mL amphotericin B at 1 × 10^6^ cells/mL. Preserved human BAL cells and blood cells were rapidly thawed at 37 °C, resuspended in RPMI-10, and centrifuged at 300× *g* for 10 min. Supernatants were discarded and the cell pellets depleted of dead/dying cells using a dead cell removal kit (Miltenyi Biotec; Bergisch Gladbach, Germany) in conjunction with MACS MS separation columns (Miltenyi Biotec) according to the manufacturer’s instructions. Total viable cells were quantified and resuspended at 1 × 10^6^ cells/’mL in RPMI-10 with additives as above. All cell types (RAW 264.7, lung, and blood) were plated in 12-well tissue culture plates at 1 mL/well. Cultured cells were then exposed to final concentrations of 1 or 5 μM Staurosporine (Millipore Sigma; Burlington, MA, USA), or 10, 100, or 1000 ng/mL LT, ET, or LT/ET for 3 h at 37 °C [41]. RAW 264.7 cells were then harvested by a combination of Accutase and repeated pipetting. Lung and blood cells were harvested by repeated pipetting only. All cells were centrifuged at 300× *g* for 5 min, washed once with 1X PBS, and resuspended in 100 μL of PBS. For the measurement of necrosis, each sample was then stained with Zombie Aqua fixable viability dye (BioLegend; San Diego, CA, USA) for 30 min on ice following the manufacturer’s instructions. Cells were then washed once with Cell Staining Buffer (BioLegend; San Diego, CA, USA) and resuspended in 100 μL of Annexin V Binding Buffer (BioLegend). For the measurement of apoptosis, PE-labeled Annexin V (BioLegend; San Diego, CA, USA) was next added to each sample, and cells incubated on ice an additional 30 min. All samples then underwent FcR blocking, antibody staining for cell identification, and fixation for flow cytometry as described for anthrax receptor surface expression (Table S1). 

### 4.8. Toxin Effects on B. anthracis Spore Internalization

*B. anthracis* spores were labeled with pHrodo iFL Red STP ester amine-reactive dye (Invitrogen; Waltham, MA, USA) per the manufacturer’s instructions and lyophilized in aliquots for subsequent use. RAW 264.7 cells were plated at 37 °C the day prior to experiments in 12-well tissue culture plates at 1 × 10^6^ cells/well in RPMI-10 with additives. Plating medium was replaced with fresh medium on the day of the experiment. Preserved lung and blood cells were thawed, prepared, and plated at 1 × 10^6^ cells/mL on the day of the experiment as described for apoptosis and necrosis induction. Cultured cells were then exposed to final concentrations of 10, 100, or 1000 ng/mL LT, ET, or LT/ET for 3 h at 37 °C. pHrodo-labeled *B. anthracis* spores were then added at 10 spores/cell in 50-μL aliquots of RPMI-10. As a negative control for actin-mediated particle internalization, some cells were exposed to 5 μM Cytochalasin D (Millipore Sigma; Burlington, MA, USA) 1 h prior to spore addition. Spore exposure was allowed to proceed for 30 or 120 min at 37 °C. At each time point, cells were harvested by Accutase and repeated pipetting (RAW 264.7 cells) or pipetting alone (lung and blood cells). All cells were centrifuged at 300× *g* for 5 min, washed once with 1X PBS, and resuspended in 100 μL of PBS. Samples were then stained with Zombie Aqua fixable viability dye (BioLegend; San Diego, CA, USA) for 30 min on ice, followed by FcR blocking, antibody staining for cell identification, and fixation for flow cytometry as described for anthrax receptor surface expression (Table S1).

### 4.9. Statistical Analysis

All statistical analyses were performed using Prism 8.0 (GraphPad Software; San Diego, CA, USA). Where applicable, results are depicted as mean + SEM. Differences in toxin receptor expression and PA binding were analyzed by one-way analysis of variance (ANOVA) with Tukey’s multiple comparisons test. Differences in apoptosis and necrosis were determined by one-sample single comparison t tests comparing each treatment to the untreated control. As the spore internalization data did not have a normal distribution, and to minimize the effects of outliers, differences in this data were analyzed by non-parametric Friedman tests with Dunn’s multiple comparisons test. A *p*-value < 0.05 was considered significant in all statistical tests performed.

## Supplementary Materials

The following are available online at https://www.mdpi.com/2072-6651/12/7/464/s1, Table S1: Antibodies and dyes used for flow cytometry, Figure S1: PA Binding Comparisons, Figure S2: Apoptosis Comparisons, Figure S3: Necrosis Comparisons.
