# Supplementary material for: Anthrax Edema and Lethal Toxins Differentially Target Human Lung and Blood Phagocytes
Source: Toxins (Basel). 2020 Jul 20;12(7):464. doi: 10.3390/toxins12070464 (PMC7405021; doi:10.3390/toxins12070464)
Supplement: Supplementary file 1 [file toxins-12-00464-s001.pdf]

Supplementary Materials: Anthrax Edema and Lethal Toxins Differentially Target Human Lung and Blood Phagocytes

Vineet I. Patel, J. Leland Booth, Mikhail Dozmorov, Brent R. Brown and Jordan P. Metcalf

Table S1. Antibodies and dyes used for flow cytometry.

Marker	Dye	Clone	Company	Catalog #	General Assay			
					TEM8/CMG2 Surface Expression	PA Binding	Apoptosis/Necrosis Induction	Spore Uptake
BDCA1	PerCP-Cy5.5	L161	BioLegend	331514	+	+	+	+
HLA-DR	Pacific Blue	L243	BioLegend	307633	+	+	+	+
Langerin	APC	10E2	BioLegend	352206	+	−	+	+
Langerin	PE	10E2	BioLegend	352204	−	+	−	−
CD3	PE	HIT3a	BioLegend	300308	−	+	−	−
CD3	PE-Cy5	HIT3a	BioLegend	300310	+	+	+	+
CD3	APC	HIT3a	BioLegend	300312	+	−	+	+
CD11c	PE-Cy7	3.9	BioLegend	301608	+	+	+	+
CD14	AF700	HCD14	BioLegend	325614	+	+	+	+
CD19	AF488	HIB19	BioLegend	302219	+	+	+	+
CD19	PE-Cy5	HIB19	BioLegend	302210	+	+	+	+
CD20	PE-Cy5	2H7	BioLegend	302308	+	+	+	+
CD56	PE-Cy5	HCD56	BioLegend	318308	+	+	+	+
Human TEM8	PE	200C1339	Novus Biologicals	NB100-56585PE	+	−	−	−
Mouse TEM8	Unlabeled	polyclonal	Novus Biologicals	NBP1-77233	+	−	−	−
Human CMG2	PE	polyclonal	Novus Biologicals	FAB2940P	+	−	−	−
Mouse CMG2	Unlabeled	polyclonal	Novus Biologicals	NBP1-68911	+	−	−	−
Donkey anti-rabbit	PE	polyclonal	BioLegend	406421	+	−	−	−
Protective Antigen	AF-647	n/a	Molecular Probes	A30009	−	+	−	−

Necrosis	Zombie Aqua	n/a	BioLegend	423102	+	+	+	+
Apoptosis	PE	Annexin V	BioLegend	640947	−	−	+	−

"+" = Indicated reagent was used in assay, "−" = Indicated reagent was not used in assay.

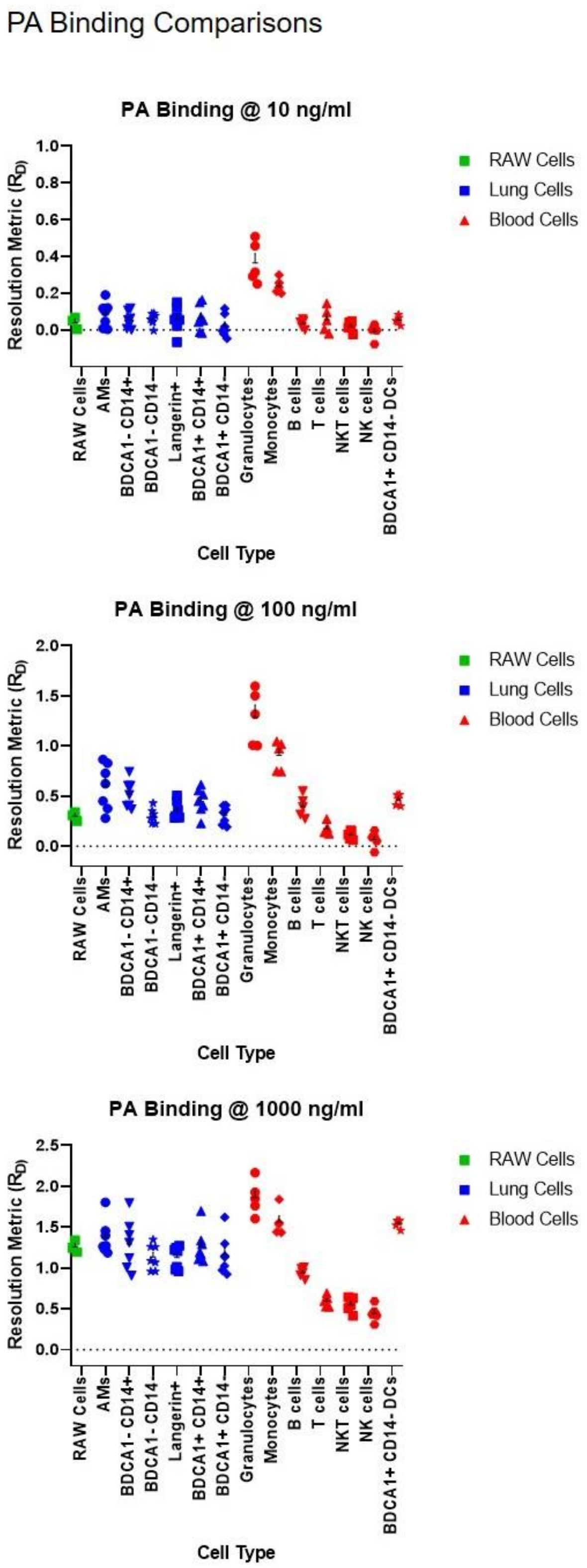

Figure S1. Blood granulocytes and monocytes bind/internalize the most PA at all exposure concentrations. Comparisons of AF-647-labeled PA binding to RAW 264.7 (green), AARP subsets (blue), and blood subsets (red) at 10 (top), 100 (middle), and 1000 ng/mL (bottom) after 30 min incubation. Graphs represent mean + SEM with individual results overlaid.

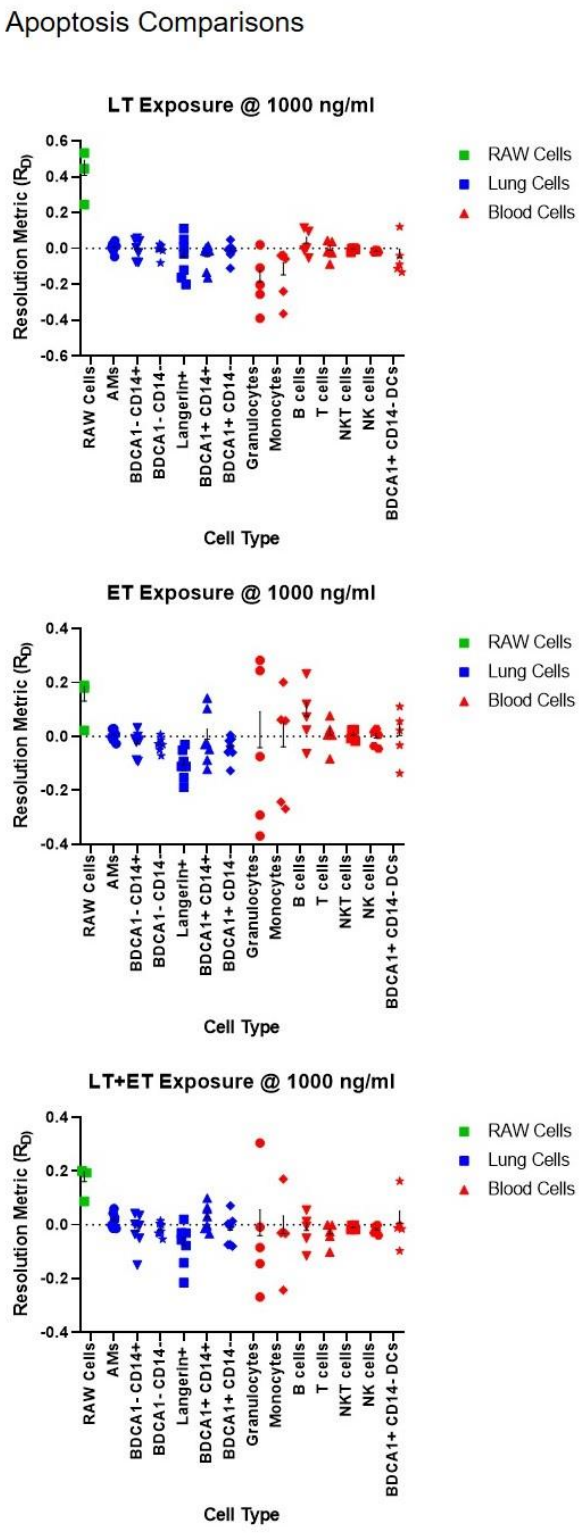

Figure S2. Anthrax lethal toxin only induces apoptosis in the mouse macrophage RAW 264.7 cell line. Comparisons of apoptosis in RAW 264.7 cells (green), AARP subsets (blue), and leukocyte subsets (red) at 1000 ng/mL LT, ET, or LT+ET after 3 h toxin exposures. Graphs represent mean + SEM with individual results overlaid.

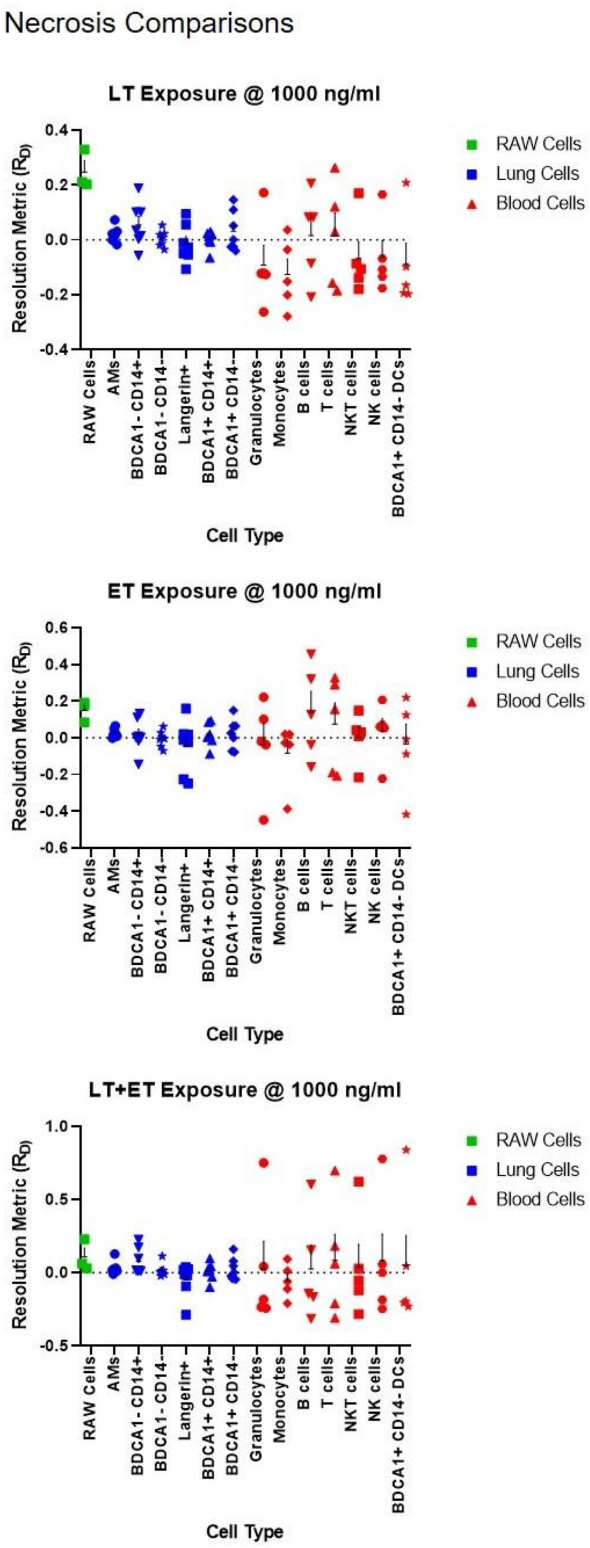

Figure S3. Anthrax LT, and to a lesser extent ET, only induce necrosis in the mouse macrophage RAW 264.7 cell line. Comparisons of necrosis in RAW 264.7 cells (green), AARP subsets (blue), and leukocyte subsets (red) at 1000 ng/mL LT, ET, or LT+ET after 3 h toxin exposures. Graphs represent mean + SEM with individual results overlaid.
